# Light-activated cAMP signaling controls sodium-driven motility in *Vibrio cholerae*

**DOI:** 10.1073/pnas.2530860123

**Published:** 2026-04-09

**Authors:** Jun Xu, Shuichi Nakamura, Suzuna Tomoyose, Reika Shimabuku, Rintaro Tomioka, Tetsu Yamashiro

**Affiliations:** ^a^Department of Bacteriology, Graduate School of Medicine, University of the Ryukyus, Ginowan, Okinawa 901-2720, Japan; ^b^Department of Applied Physics, Graduate School of Engineering, Tohoku University, Sendai, Miyagi 980-8579, Japan; ^c^Department of Bioengineering, School of Engineering, The University of Tokyo, Tokyo 113-8656, Japan

**Keywords:** photokinesis, *Vibrio cholerae*, bacterial motility, photoactivated adenylyl cyclase, sodium-motive force

## Abstract

Light is one of the most pervasive physical cues in aquatic environments, yet its impact on nonphototrophic pathogens remains largely unexplored. Here, we show that a strain of cholera bacterium *Vibrio cholerae* directly couples illumination to motility through cyclic AMP (cAMP) signaling. Exposure to visible light rapidly elevates intracellular cAMP and increases swimming speed, whereas deletion of the single adenylyl cyclase gene (*cyaA*) abolishes both responses; complementation or addition of exogenous cAMP restores the phenotype. Heterologous expression of *V. cholerae* CyaA in an *Escherichia coli* Δ*cyaA* Δ*cpdA* background reconstitutes light-activated cAMP synthesis, indicating that CyaA confers photoreactivity. Purified CyaA exhibits a reversible light-dependent spectral shift consistent with flavin-dependent photochemistry, identifying it as a light-responsive cyclase. Illumination triggers rapid membrane hyperpolarization and sodium efflux, strengthening the sodium-motive force that powers the flagellar motor. This response persists under nutrient-limited conditions. Together, these findings define a light → cAMP → sodium-motive force coupling axis in *V. cholerae*, suggesting that ambient light may influence motility and dispersal in sunlit environments.

## Significance

Bacteria use second messengers to couple environmental cues to behavior, but how light regulates motility in *Vibrio cholerae* is not well defined. We show that in strain AJ10, a photoresponsive adenylyl cyclase (CyaA) elevates intracellular cAMP under illumination and is required for light-enhanced swimming. Light responsiveness persists under nutrient limitation, linking an ecologically relevant cue to cyclic-nucleotide signaling and sodium-powered flagellar energetics. These findings define a light–cAMP–motility axis that links environmental illumination to second messenger signaling and sodium-powered flagellar energetics in a major bacterial pathogen.

*Vibrio cholerae* is a facultative pathogen that alternates between sunlit aquatic reservoirs and the human intestine (1, 2). In coastal and estuarine water, the bacterium encounters steep, rapidly fluctuating gradients of temperature, salinity, pH, and osmolarity (3–5). To survive these transitions, *V. cholerae* dynamically regulates biofilm formation, virulence gene expression, and motility, enabling cells to locate nutrient-rich niches and to penetrate the intestinal mucus layer during infection (6, 7).

Environmental cues known to influence *V. cholerae* motility are primarily chemical (bile, amino acids, quorum signals) or mechanical (viscosity, surface contact) (7–11). Whereas light, arguably the most pervasive physical signal in shallow waters, has received little attention. Light has been recognized as an important environmental signal for many nonphototrophic bacteria, influencing biofilm formation, stress adaptation, and motility (12). In enteric species such as *Escherichia coli* and *Salmonella*, illumination has been reported to act as a chemorepellent or to alter flagellar behavior (13, 14). During a phenotypic screen of clinical and environmental isolates, however, we identified *V*. *cholerae* O1 strain AJ10, recovered from a riverine estuary in Okinawa, Japan (15), that swims markedly faster when exposed to visible light. Here, we demonstrate photokinesis mediated by a photoresponsive adenylyl cyclase in an enteric bacterium.

In many bacteria, the second messenger cyclic AMP (cAMP) coordinates carbon metabolism, virulence, and motility through the cAMP receptor protein (CRP) regulatory network (16, 17). Certain soil and freshwater species encode photo-activated adenylyl cyclases (PACs) that directly couple light to intracellular cAMP synthesis (18–20). We therefore asked whether *V. cholerae* can convert ambient light into a cAMP signal that modulates its sodium-driven flagellar motor (21, 22).

Here we show that illumination of strain AJ10 rapidly elevates intracellular cAMP, the deletion of the sole class I adenylyl cyclase gene (*cyaA*) abolishes this response and the associated motility boost, and that purified CyaA is a flavin-binding photoactivated cyclase whose spectral and kinetic properties satisfy the signs of a BLUF/LOV-type sensor (18, 23). The resulting increase in cAMP activates sodium antiporters and strengthens the sodium-motive force, thereby enhancing flagellar motor output. Notably, the light-responsive CyaA variant characterized here is not universally conserved across *V. cholerae*, comparative sequence analyses indicate that AJ10-like N-terminal signatures occur in a subset of strains. Together, these findings reveal a previously unrecognized photon-to-motility signaling pathway in an enteric pathogen and suggest that daylight may act as an ecological cue shaping *V. cholerae* AJ10-type strains dispersal and transmission.

## Results

### Light Enhances Motility and cAMP Production in *V. cholerae*.

To quantify light-dependent changes in motility, we recorded dark-field videos at 60 fps for 10 to 20 s and extracted single-cell trajectories using ImageJ TrackMate (24). Trajectories shorter than 1 s were excluded; instantaneous speed was calculated from frame-to-frame displacement, and motile cells were defined using a speed-based threshold (*SI Appendix*, *Methods*).

**Fig. 1.**
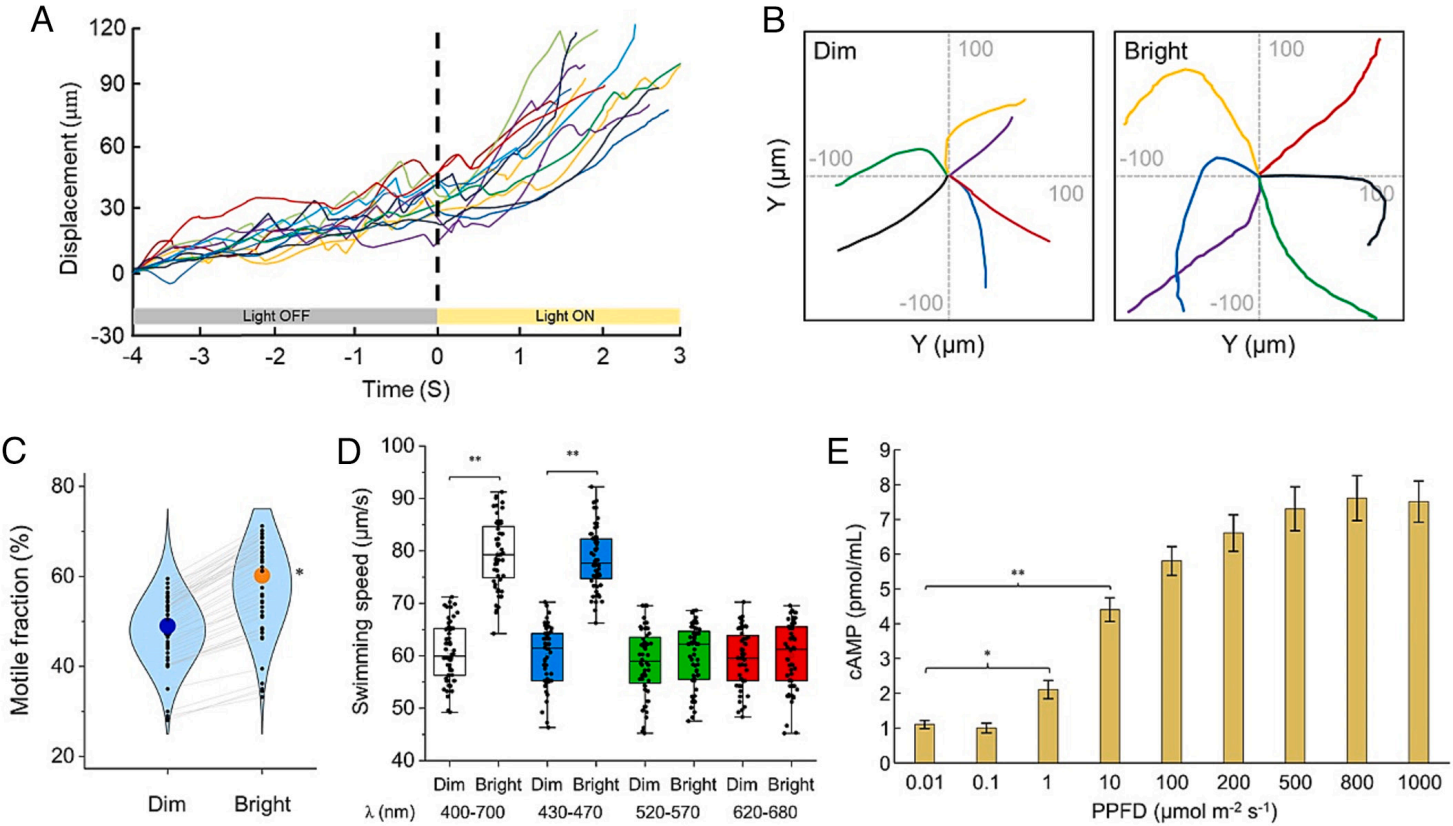
Visible light increases *V. cholerae* AJ10 motility and elevates intracellular cAMP. (*A*) Time course of displacement for representative single-cell trajectories during a dark-to-light transition (dim: 0.01 μmol m^−2^ s^−1^ and bright: 100 μmol m^−2^ s^−1^). Illumination was switched on at t=0 s (dashed line). Each colored trace represents one tracked cell; gray/yellow bars indicate light OFF/ON intervals. (*B*) Representative trajectories plotted over 2 s for the dim and bright conditions (same duration for both panels), shown at the same spatial scale. (*C*) Motile fraction in dim vs bright conditions quantified per field of view (FOV). Small black dots represent individual FOV values; gray lines connect paired measurements from the same FOV before and after illumination. Large colored circles indicate the mean across FOVs for each condition from three biological replicates (15 FOVs per replicate). Statistical significance was assessed using a two-tailed paired Student’s *t* test on biological replicate means (n = 3). Motile cells were defined using a speed threshold criterion (*SI Appendix*, *Methods*). (*D*) Swimming speed under illumination with different spectral ranges. Box plots show distributions of single-cell swimming speeds; dots represent individual trajectories. The indicated wavelength bands were selected using band-pass filters (400 to 700 nm, 430 to 470 nm, 520 to 570 nm, and 620 to 680 nm). Differences across spectral conditions were assessed by one-way ANOVA with Tukey’s multiple comparison test on biological replicate mean speeds (each replicate mean was calculated from 15 FOVs, n = 3 biological replicates). (*E*) Intracellular cAMP levels measured as a function of light intensity (PPFD). Differences across light intensity conditions were assessed by one-way ANOVA with Tukey’s test on biological replicate means (n = 3).

Illumination triggered a rapid enhancement of motility in *Vibrio* cells. Displacement analysis of individual trajectories revealed that cells accelerated immediately after the onset of light exposure (Fig. 1*A* and Movie S1). Swimming tracks over the same time window further demonstrated longer displacements in the illuminated state compared with dim conditions (Fig. 1*B*). Quantification across multiple fields of view showed that the fraction of motile cells increased significantly under bright illumination (Fig. 1*C*), indicating that light not only enhanced the performance of already motile cells but also recruited previously quiescent cells into active swimming. We next examined whether this enhancement was wavelength dependent. Swimming speed increased significantly under broadband white light (400 to 700 nm) and blue light (430 to 470 nm), whereas no significant change was observed under green (520 to 570 nm) or red (620 to 680 nm) illumination (Fig. 1*D* and Movies S1–S4). These results suggest that blue wavelengths are most effective in stimulating motility.

Given the rapid onset of motility following light exposure, we next sought to identify intracellular signaling molecules that might mediate this response. In many bacteria, second messengers such as cyclic AMP (cAMP) are known to modulate behaviors including chemotaxis, surface attachment, and flagellar regulation (25–27). Because previous studies implicated light-sensitive adenylyl cyclases in bacterial photobehavior (18, 23, 28), we asked whether light modulates intracellular cAMP levels in these cells. Indeed, cAMP concentrations increased in a light intensity–dependent manner, with saturation occurring above ~500 µmol m^−^^2^ s^−^^1^ photosynthetic photon flux density (PPFD) (Fig. 1*E*). Unless otherwise noted, motility recordings were performed at PPFD values in the ~100 to 200 µmol m^−^^2^ s^−^^1^ range (with dim controls <10 µmol m^−^^2^ s^−^^1^), matching the intensities used for the light-gradient assays, which are within reported ranges for shallow/turbid coastal waters (29, 30). Together, these results indicate that *V. cholerae* AJ10 strain exhibits a blue light-induced motility enhancement, and a light-sensitive adenylyl cyclase may be involved.

### Illumination Enhances Speed and Introduces a Weak Directional Component.

**Fig. 2.**
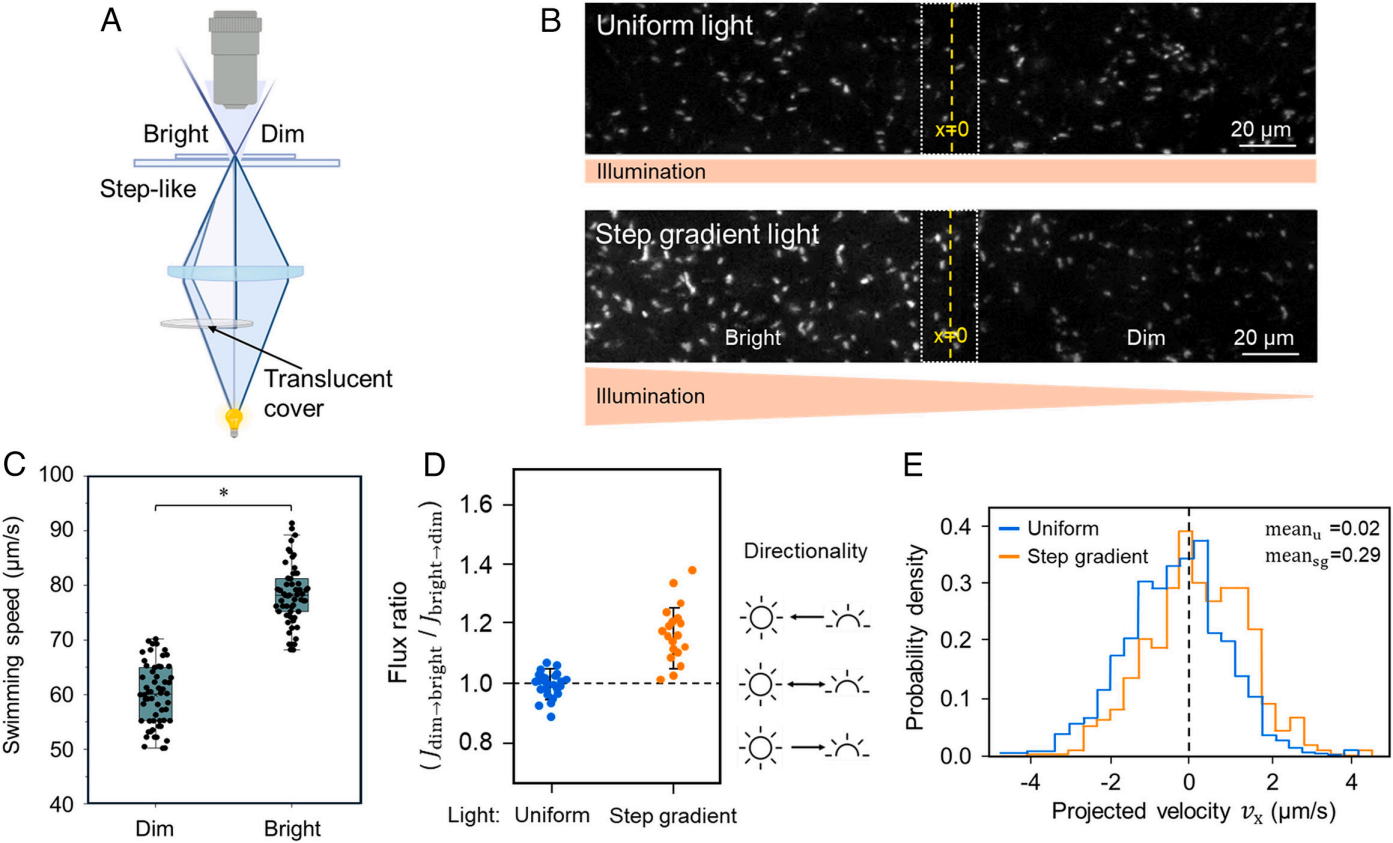
Step-like light gradient assay reveals light-enhanced swimming speed and a modest directional bias across an illumination boundary. (*A*) Schematic of the step gradient illumination setup. A translucent cover placed in the illumination path generates a step-like light field with adjacent bright/dim regions in the imaging plane. (*B*) Representative images under spatially uniform illumination (*Top*) and after switching to step gradient illumination (*Bottom*; only the *Left* area was illuminated). The dashed white box denotes a 20 µm boundary zone used for analyses, and the yellow dashed line marks the boundary center (x = 0). (*C*) Swimming speed of motile cells measured in the dim and bright regions under step gradient illumination. Boxes indicate the interquartile range with the center line showing the median. Dots represent individual cells from three independent trials (n = 3). Statistical significance was assessed using two-tailed paired Student’s *t* test on trial-mean speeds (*P* < 0.05). (*D*) Boundary-crossing flux asymmetry quantified as the flux ratio $J_{dim\to bright}/J_{bright\to dim}$ under uniform and step gradient illumination. Each dot represents one movie; for inference, movie-level values were averaged within each biological replicate and comparisons between illumination conditions were performed across biological replicates (n = 3). The dashed line indicates the symmetric flux (ratio = 1). Icons at the *Right* illustrate the expected flux direction. (*E*) Net drift along the gradient axis quantified by the distribution of projected velocity $v_{x}$ (positive indicates motion toward the bright/*Left* region). The histograms show $v_{x}$ distributions under uniform (blue) and step gradient (orange) illumination. The dashed line marks $v_{x}=0$ (no drift). Mean projected velocities are indicated (mean_u_, uniform; mean_sg_, step gradient).

Microbial behaviors controlled by light generally fall into two categories: phototaxis, directional movement toward or away from a light source, and photokinesis, a change in motility that depends on light intensity but is not inherently directional (31–33). To evaluate whether the light response in *V. cholerae* includes any directional component in addition to speed modulation, we established a bright/dim boundary assay that generates a step-like illumination profile within a single field of view (Fig. 2 *A* and *B* and Movie S5). Under step gradient illumination, cells in bright regions exhibited swimming speeds significantly higher than those in the dim regions (Fig. 2*C*). Segmentation-based analysis further indicated that total cell density was broadly similar across the field, whereas the fraction of motile cells increased toward the bright side (*SI Appendix*, Fig. *S1*). Such spatial redistribution under light patterns has been widely analyzed in photokinetic systems, where speed modulation alone can shape apparent density profiles (34).

To test whether light biases the *V. cholerae* swimming direction, we quantified the bacterial population flux (*J*) along a light gradient. The analysis showed that *J* from dim to bright ($J_{dim\to bright}$) exceeded the reversed one ($J_{bright\to dim}$), i.e., $J_{dim\to bright}/J_{bright\to dim}>1$ understep gradient illumination, whereas $J_{dim\to bright}/J_{bright\to dim}\approx 1$ under uniform illumination (Fig. 2*D*). We also determined drift speeds along the light gradient by projecting individual bacterial velocities onto the x-axis ($v_{x}$; where positive values indicate the “brightward” direction). While the distribution of $v_{x}$ under uniform illumination was nearly symmetric, it exhibited a positive shift under step gradient illumination (Fig. 2*E*). Together, these results show that visible light robustly enhances swimming speed (photokinesis) and can be accompanied by a modest brightward bias consistent with a weak phototactic component.

### CyaA Is Essential for Light-Induced cAMP Production and Motility Boost.

**Fig. 3.**
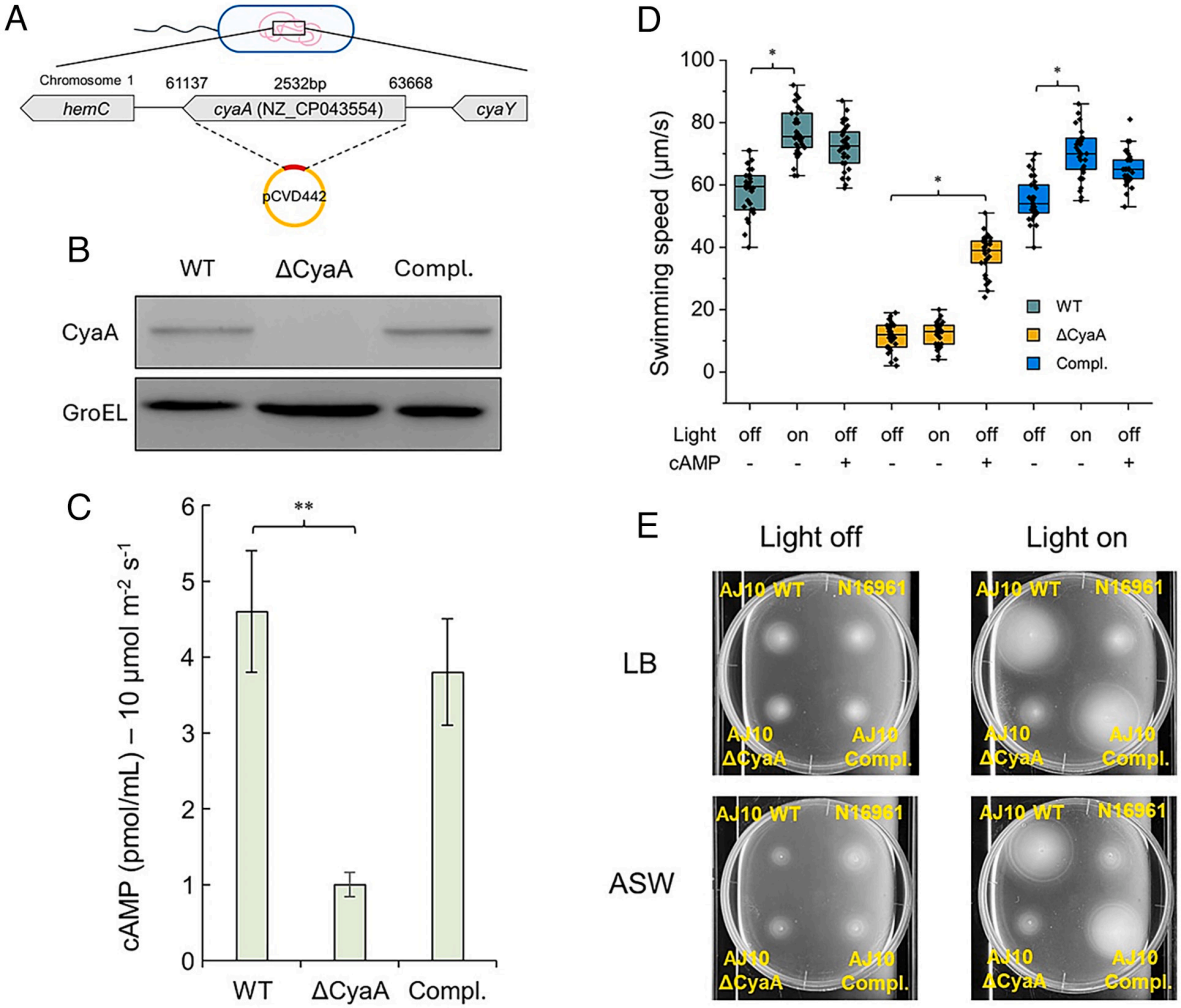
The adenylate cyclase CyaA is required for light-induced cAMP production and motility enhancement. (*A*) Schematic of *cyaA* deletion strategy. The *cyaA* locus on chromosome I was replaced with a suicide plasmid (pCVD442) via homologous recombination. (*B*) Immunoblot confirming loss of CyaA expression in the Δ*cyaA* mutant and restoration in the complemented strain (Compl.). GroEL served as a loading control. (*C*) Intracellular cAMP levels measured by ELISA in WT, Δ*cyaA*, and complemented strains after light stimulation (10 µmol m^−2^ s^−^^1^, 60 s). Data are from three replicates, *P* < 0.01 (Student’s *t*test). (*D*) Swimming speed of WT, Δ*cyaA*, and complemented strains in the dark and under illumination, with or without exogenous cAMP supplementation (1 mM). Box plots show interquartile ranges, medians, 10th to 90th percentiles, and individual data points. *P* < 0.05 (ANOVA with Tukey’s test). (*E*) Soft agar motility assay under light-off and bright conditions.

To determine whether the adenylyl cyclase CyaA mediates the light-dependent increase in cAMP and motility, we constructed a *cyaA* deletion mutant in the photokinetic strain AJ10. The *cyaA* locus was replaced by homologous recombination, and loss of CyaA expression was confirmed by immunoblotting (Fig. 3 *A* and *B*). Because sequence features implicated in photoreactivity are not present in all *V. cholerae* genomes, our mechanistic analyses focus on the AJ10-type CyaA allele.

As shown above, Illumination of wild-type (WT) cells induced a rapid rise in intracellular cAMP, whereas the Δ*cyaA* mutant showed only a basal level. Complementation fully restored the light-dependent response (Fig. 3*C*). These results demonstrate that CyaA is required for photoactivated cAMP synthesis.

Consistent with this, the swimming speed of the Δ*cyaA* strain remained low and unresponsive to illumination, in contrast to the clear light-dependent boost observed in the WT and complemented strains with increased swimming speed by ~25 to 30 % (Fig. 3*D* and Movies S6 and S7). Normalized swimming speeds of each strain to their own dark-condition baselines clearly revealed that only WT and complemented cells exhibited a substantial fold increase in motility under light, while Δ*cyaA* cells showed negligible change (*SI Appendix*, Fig. S2). Supplementation of the medium with exogenous cAMP rescued the motility phenotype of the Δ*cyaA* mutant, confirming that cAMP acts downstream of CyaA to enhance motility (Movie S8).

For nutrient-limited conditions, cells growing to OD_600_ ≈ 0.5 in LB were collected, washed, and resuspended in ASW (artificial seawater) at matched cell density. Growth in soft agar was evaluated under identical light settings in bright versus dim illumination (*SI Appendix*, *Methods*). Consistent with the single-cell data, soft agar assay showed greater expansion for illuminated WT and complemented strains compared with Δ*cyaA* and the nonphotokinetic strain N16961 (Fig. 3*E*). The light-induced expansion persisted not only in nutrient-rich LB but also in nutrient-limited ASW medium, indicating that the photokinetic effect is not dependent on cell growth. The ability of *V. cholerae* AJ10 to sustain light-responsive motility under low-nutrient conditions suggests that this mechanism could operate under ecologically relevant energy states. Together, these results demonstrate that the adenylate cyclase CyaA from AJ10 is indispensable for light-induced cAMP production and subsequent enhancement of motility.

### CyaA Confers Light Responsiveness and Exhibits a Flavin Photocycle.

**Fig. 4.**
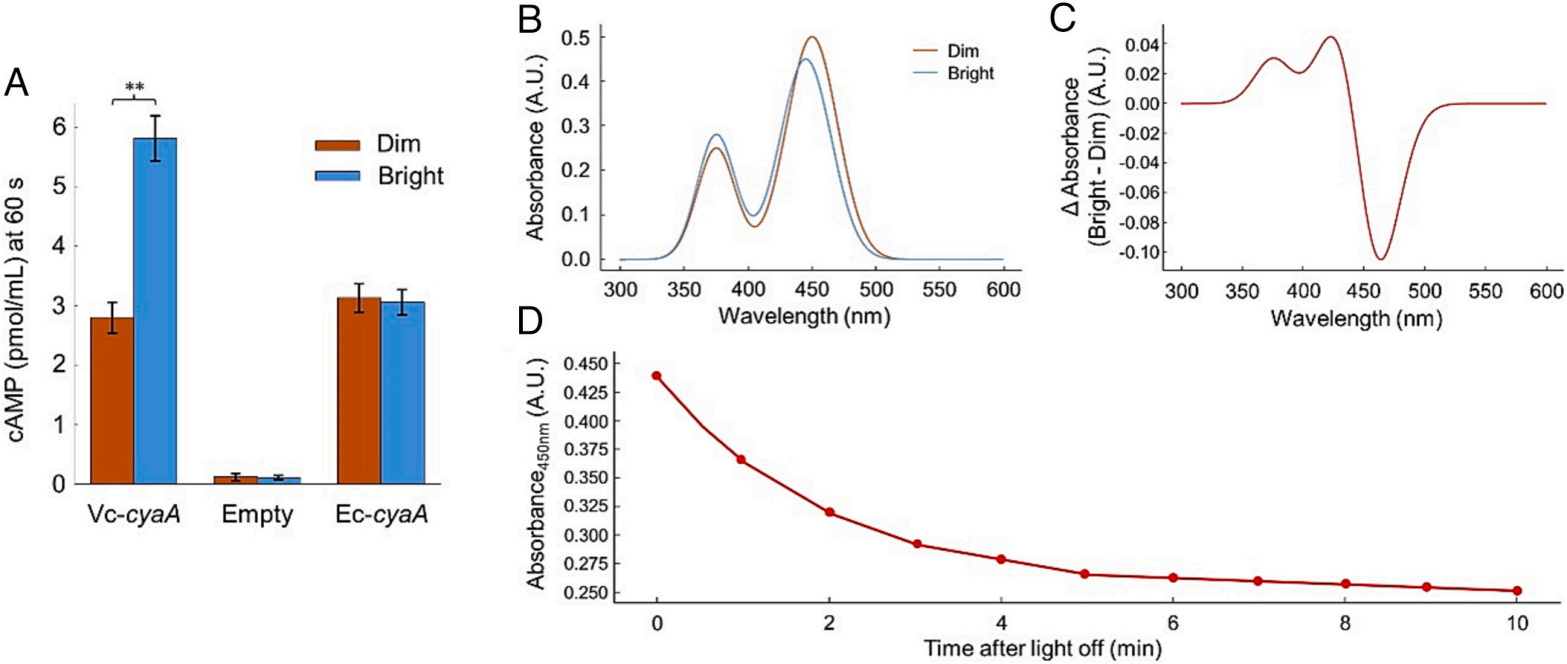
Light responsiveness of *V. cholerae* AJ10 CyaA and its photochemical properties. (*A*) cAMP production in *E. coli* Δ*cyaA* Δ*cpdA* expressing AJ10 *cyaA* (Vc-*cyaA*), *E. coli* *cyaA* (Ec-*cyaA*), or empty vector, measured under dim and bright conditions (200 µmol m^−2^ s^−^^1^, 60 s). Bars show mean ± SD of three independent biological replicates. Statistical significance between dim and bright conditions within each construct was assessed using a two-tailed paired Student’s *t* test on replicate values (n = 3). (*B*) UV–visible absorption spectra of purified His-tagged Vc-CyaA under dim and bright conditions. (*C*) Light-minus-dark difference spectrum of purified Vc-CyaA derived from the spectra in (*B*). (*D*) Recovery kinetics of the 450 nm absorbance after cessation of illumination, monitored every 30 s for 10 min. data were fitted to a single-exponential decay (τ_1/2_ = 2.1 min, *r*^2^ = 0.99). Traces are representative of three independent experiments.

To test whether CyaA from AJ10 alone is sufficient to mediate light responsiveness, we expressed the AJ10 *cyaA* gene in the adenylyl cyclase/phosphodiesterase-null host *E. coli* MG1655 Δ*cyaA* Δ*cpdA*. We verified that AJ10 *cyaA* expression did not produce obvious growth or Na^+^/pH stress phenotypes in the *E. coli* host under the induction conditions used. Upon illumination, intracellular cAMP levels increased significantly compared with the dark condition, whereas cells carrying the empty vector or the native *E. coli* *cyaA* showed no light-dependent change (Fig. 4*A*). These results demonstrate that CyaA can confer a photoresponsive cAMP pathway in a heterologous host.

To investigate the underlying photochemical basis, purified His-tagged CyaA was analyzed by UV–visible absorption spectroscopy. The protein displayed characteristic flavin peaks near 375 and 450 nm, which decreased in amplitude upon illumination (Fig. 4*B*). Subtraction of light and dark spectra yielded a difference spectrum with a positive band at 375 nm and a negative band at 450 nm (Fig. 4*C*). After cessation of illumination, the 450 nm absorbance recovered monoexponentially with a half-time of approximately 3 min and returned close to the dark baseline within 10 min (Fig. 4*D*).

Domain-swap analysis between the light-responsive AJ10 CyaA and the nonresponsive N16961 homolog further supported these findings (*SI Appendix*, Fig. S3). Chimeric constructs combining the N-terminal regions showed that domains contribute to full light-dependent activity, as replacement of either region reduced the amplitude of the cAMP response. Together, these results establish that CyaA from AJ10 functions as a photoactivated adenylyl cyclase in which coordinated elements of the terminal regions couple light absorption to catalytic activation of cAMP synthesis.

### Light-Induced cAMP Enhances Motility Through Sodium–Membrane Potential Coupling.

In *V. cholerae*, the rotation of the polar flagellum is powered by the sodium-motive force (SMF), an electrochemical gradient composed of membrane potential (Δψ) and the sodium concentration gradient (ΔμNa^+^) across the cell membrane (SMF = Δψ + ΔμNa^+^) (21, 35, 36). Previous studies have shown that changes in SMF can directly influence flagellar torque and swimming behavior in marine and halophilic bacteria (22, 37, 38). Given the observed role of cAMP in light-enhanced motility, we hypothesized that cAMP might stimulate sodium export or ion channel activity, thereby boosting SMF and energizing the motor.

**Fig. 5.**
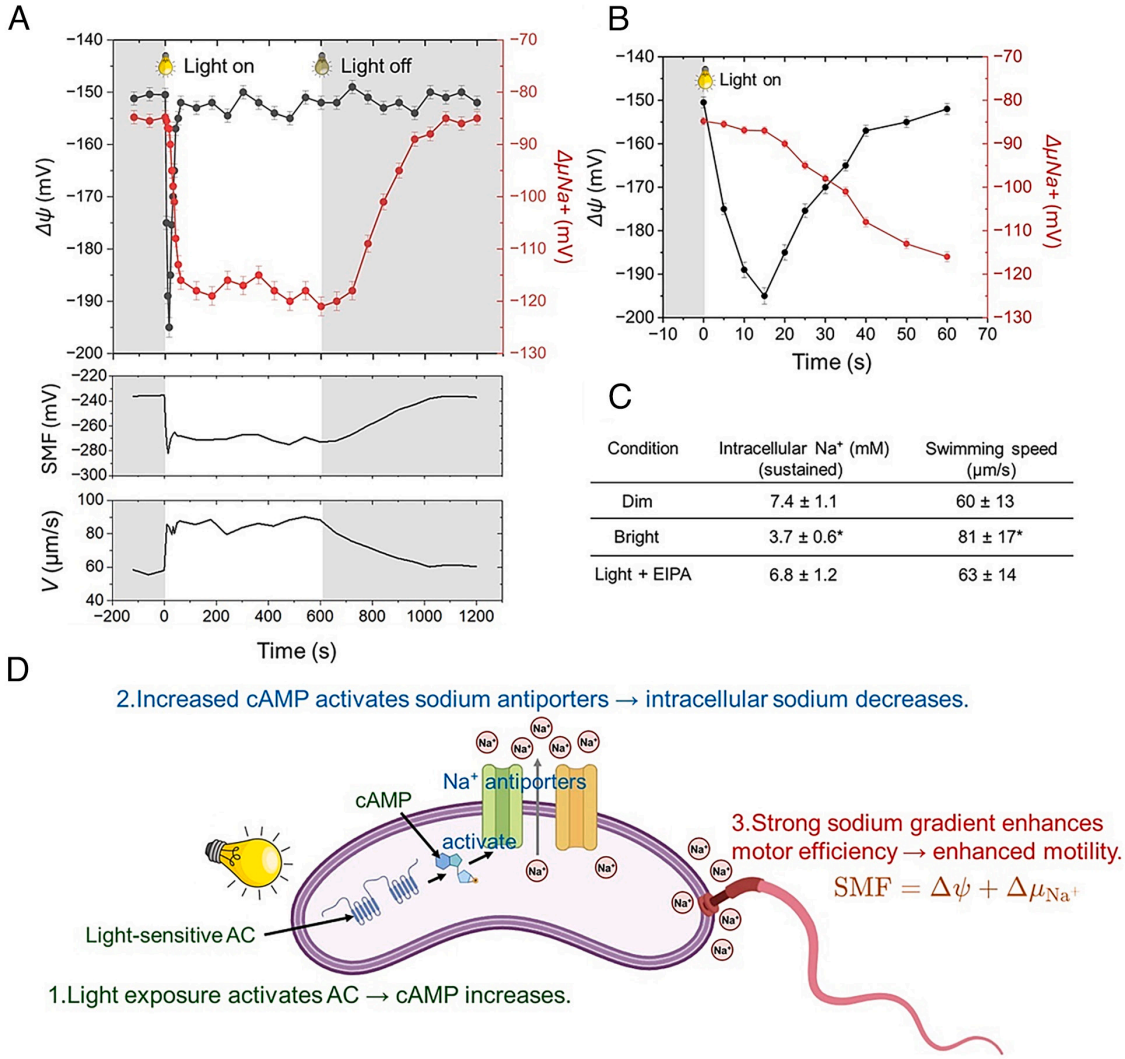
Light-induced cAMP regulates intracellular sodium and membrane potential to enhance motility. (*A*) Simultaneous measurement of membrane potential (Δψ, black) and sodium potential (ΔμNa^+^, red) during light on/off cycles. The *Top* panel shows mean traces with shaded light period; *Middle* and *Lower* panels show corresponding changes in sodium-motive force (SMF) and swimming speed (V). (*B*) Expanded view of the initial 60 s after illumination onset showing rapid hyper/depolarization (Δψ) and rise of ΔμNa^+^. (*C*) Intracellular sodium concentrations and corresponding swimming speeds under dim, bright, and light + EIPA (Na^+^/H^+^ antiporter inhibitor) conditions. Data are presented as mean ± SD of three replicates. *P* < 0.05 compared with dim (Student’s *t*test). (*D*) Schematic model summarizing the proposed mechanism. Light activates the photosensitive adenylyl cyclase (AC), increasing cAMP, which in turn activates Na^+^ antiporters and lowers intracellular Na^+^ concentration. The resulting increase in sodium-motive force strengthens flagellar motor output and enhances swimming speed.

We simultaneously monitored the two principal components of the sodium-motive force (SMF), membrane potential (Δψ) and the Na^+^ electrochemical potential (ΔμNa^+^), using the fluorescent probes DiSC3(5) and SBFI-AM, respectively. Fluorescence was collected as time courses during alternating dark/light periods, and probe signals were converted to Δψ and ΔμNa^+^ values using calibration procedures (*SI Appendix*, *Methods*). Upon light exposure, cells exhibited an immediate hyperpolarization of Δψ, shifting from approximately −150 to −190 mV within seconds (Fig. 5 *A* and *B*, black curve). This response was transient and relaxed toward baseline within ~30 s. In contrast, ΔμNa^+^ increased with a delay but continued to rise during illumination (Fig. 5 *A* and *B*, red curve), producing a sustained increase in SMF that paralleled the enhancement of swimming speed (Fig. 5 *A*, *Lower* panels). The enlarged 0 to 60 s view (Fig. 5*B*) highlights this temporal decoupling: Δψ changes sharply and briefly, whereas ΔμNa^+^ strengthens more gradually and persists. Together, these kinetics indicate that light stimulation rapidly perturbs the electrical component of SMF and subsequently promotes a longer-lasting increase in the Na^+^-coupled driving force, consistent with the time course of motility enhancement.

To determine whether sodium transport contributes to this process, intracellular Na^+^ concentrations were quantified under dim and bright conditions. Light exposure reduced intracellular Na^+^ from 7.4 ± 1.1 mM to 3.7 ± 0.6 mM, coinciding with a significant increase in swimming speed (Fig. 5*C*). Treatment with the Na^+^/H^+^ antiporter inhibitor EIPA prevented both the sodium decrease and the motility enhancement, indicating that light-induced cAMP production activates Na^+^ export through antiporters.

Together, these observations support a model in which photoactivation of adenylyl cyclase elevates intracellular cAMP, leading to the activation of Na^+^/H^+^ antiporters and a reduction of cytoplasmic Na^+^ concentration. The resulting increase in sodium-motive force strengthens flagellar motor performance, producing the observed motility boost under illumination (Fig. 5*D*).

## Discussion

Natural light gradients structure aquatic habitats and covary with temperature, oxygenation, and nutrient flux (6, 29, 39, 40). While phototrophic microbes exploit photons for energy and symbiotic vibrios detect light to regulate bioluminescence (41, 42), evidence has remained limited for how nonphototrophic bacteria use light as a behavioral cue. Light can shape the physiology of diverse heterotrophs (12), and in enteric species such as *E. coli* and *Salmonella*, illumination can act as a chemorepellent or influence flagellar motion (13, 14, 43). Here, we show that *V. cholerae* O1 strain AJ10 senses visible light through a photoresponsive adenylyl cyclase, converts illumination into a rapid cAMP surge, and thereby strengthens sodium-powered motility-a distinct form of photokinesis in an enteric pathogen.

Three independent lines of evidence identify the class I adenylate cyclase CyaA as the sensor-effector mediating this response. Deletion of *cyaA* abolished light-induced cAMP accumulation and motility enhancement, whereas chromosomal complementation or exogenous cAMP restored both phenotypes. Expression of AJ10 CyaA in an *E. coli* Δ*cyaA* Δ*cpdA* host produced a clear light-dependent increase in cAMP, while *E. coli* CyaA and vector controls remained unresponsive. Finally, purified CyaA exhibited a reversible flavin-dependent photocycle, with illumination causing a decrease near 450 nm and an increase near 375 nm, followed by thermal recovery within minutes. Together, these features support classification of AJ10 CyaA as a photoactivated adenylyl cyclase (PAC) capable of directly converting light into cAMP signaling.

Because AJ10-like N-terminal signatures are not uniformly conserved across all *V. cholerae* (*SI Appendix*, Fig. S4), the ecological scope of this mechanism is likely lineage dependent. One possible explanation is that light-responsive motility is advantageous primarily in shallow, sunlit aquatic microhabitats where irradiance changes sharply across small spatial scales (e.g., near the air–water interface, biofilm surfaces, or particle-associated niches). In such locations, coupling illumination to cAMP could help tune Na^+^-driven motility to promote rapid dispersal or relocation across microhabitat boundaries. Conversely, in lineages that more often occupy turbid, deeper, or host-associated niches where light cues are attenuated, the selective pressure to retain a photoactivated cyclase may be reduced, allowing loss or divergence of the light-responsive module. The patchy distribution of AJ10-like features therefore suggests ecological specialization rather than a universally conserved signaling pathway. We therefore present potential links to dispersal and host encounter as testable hypotheses rather than established effects on transmission. Domain-swap analysis between the light-responsive AJ10 CyaA and the nonresponsive N16961 homolog further indicates that terminal regions are required for full photoactivity (*SI Appendix*, Fig. S3). The absence of canonical BLUF or LOV motifs suggests that CyaA may employ a highly diverged flavin-binding module or an atypical chromophore pocket, potentially involving an AJ10-specific N-terminal extension (*SI Appendix*, Fig. S5) (44, 45).

Our measurements define a mechanistic cascade—light → cAMP → membrane hyperpolarization → Na^+^ efflux → increased sodium-motive force → faster flagellar rotation-linking photoperception to the electrochemical gradient that powers motility (46). The response includes a rapid Δψ change followed by a slower shift in ΔμNa^+^, consistent with immediate torque enhancement coupled to subsequent ionic adjustment. Because sodium-driven motors are widespread among marine vibrios, SMF-based tuning could be broadly useful in lineages that encode photoactivated cyclases, although its prevalence and physiological impact are likely strain dependent (47, 48).

Light-induced motility enhancement persisted under nutrient limitation, indicating that the phenotype is not restricted to energy-replete media. Upon transfer from LB into artificial seawater or HEPES-saline buffer, baseline swimming speeds declined as expected, yet illumination still elicited a measurable increase (*SI Appendix*, Fig. S6). Moreover, because natural photic zones often contain sharp spatial light gradients, a modest directional bias under step gradient illumination could, in principle, further influence how cells traverse and accumulate across microhabitat boundaries in nutrient-poor waters. The response gradually weakened with prolonged starvation but remained detectable for more than 1 h, consistent with prior observations that marine *Vibrio* can retain motility for extended periods without nutrients (49, 50). Thus, the light-dependent motility system can operate under environmentally relevant energy states and may provide an advantage during transient light exposure in nutrient-poor surface waters.

A simple physical model suggests that the observed speed increase could raise encounter rates and reduce traversal times in aquatic microenvironments (*SI Appendix*, Fig. S7 and section 1), supporting the plausibility of ecological effects on interactions with particulate matter, chitinous debris, or planktonic hosts that serve as *V. cholerae* reservoirs (51, 52). In addition, the photon flux densities used here (~100 to 200 µmol m^−2^ s^−^^1^; dim controls <10 µmol m^−^^2^ s^−^^1^) fall within ranges encountered in shallow coastal waters, particularly in turbid estuaries where light attenuates steeply with depth (29, 30). Together, these observations indicate that the light → cAMP → motility coupling described here is compatible with realistic photic-zone exposure; whether it contributes to seasonal cholera dynamics remains unknown and will require targeted testing under environmental and host-relevant conditions (53–56).

Within broader signaling networks, cAMP regulates multiple processes in *V. cholerae*, including carbon utilization, quorum sensing, and virulence gene expression (57–59). Light-dependent elevation of cAMP therefore provides a route to coordinate motility with downstream transcriptional programs relevant to nutrient acquisition and dispersal. Importantly, deletion of *crp* abolished both the light-induced and exogenous cAMP-induced speed increases (*SI Appendix*, Fig. S8), indicating that the motility phenotype depends on the canonical cAMP–CRP pathway rather than a CRP-independent effect of cAMP. CRP likely acts downstream to link cAMP elevations to motility control, potentially via transcriptional regulation of motor energetics or ion-transport processes.

Together, our results add light to the set of environmental cues shaping the behavior of AJ10-type *V. cholerae* and identify CyaA as a previously unrecognized photoactivated cyclase class. Future work should define the chromophore identity, action spectrum, and dose–response properties of CyaA and test the distribution and functional impact of related photokinetic modules across environmental vibrios and other enteropathogens. These studies will clarify when photokinesis is strain specific and when it represents a reusable sensory-motility strategy in sunlit aquatic niches.

## Materials and Methods

### Bacterial Strains.

The *V. cholerae* O1 AJ10 strain used in this study is part of the AJ strain set (AJ1 to AJ12) originally isolated from the Aja River (Naha–Urasoe area), Okinawa, Japan (15). The original report identified these isolates as *V. cholerae* O1, biotype El Tor, serotype Inaba, and described them as weakly pathogenic based on rabbit ileal loop assays, low intestinal adhesion, and minimal detectable cholera toxin production.

Full experimental procedures, including medium, reagents, strain construction, motility tracking, cAMP quantification, UV-visible spectroscopy, sodium-motive force measurements, and statistical analyses, are provided in *SI Appendix*. Light intensity at the specimen plane was measured with a calibrated photometer and reported as PPFD (µmol photons m^−2^ s^−^^1^); full optical setup details are provided in *SI Appendix*. All data needed to evaluate the conclusions in the paper are present in the paper and/or *SI Appendix*. The raw data of this study have been deposited in Mendeley Data, V1 (https://doi.org/10.17632/23s5y33dn9.1).

## Supplementary Material

Appendix 01 (PDF)

Movie S1. Photokinesis in *V. cholerae* (400–700 nm).

Movie S2. *V. cholerae* cells swimming in blue light (430–470 nm).

Movie S3. *V. cholerae* cells swimming in green light (520–570 nm).

Movie S4. *V. cholerae* cells swimming in red light (620–680 nm).

Movie S5. *V. cholerae* cells swimming across light gradient.

Movie S6. Swimming wide-type *V. cholerae* AJ10 cells upon light exposure.

Movie S7. Swimming *ΔCyaA* cells upon light exposure.

Movie S8. Swimming *ΔCyaA* cells with addition of cAMP in the dim.

## Data Availability

The raw data and analysis code of this study have been deposited in Mendeley Data, V1 (https://doi.org/10.17632/23s5y33dn9.1) (60).

## References

[1] J. Reidl, K. E. Klose, *Vibrio cholerae* and cholera: Out of the water and into the host. FEMS Microbiol. Rev. **26**, 125–139 (2002). 12069878 10.1111/j.1574-6976.2002.tb00605.x

[2] C. Lutz, M. Erken, P. Noorian, S. Sun, D. McDougald, Environmental reservoirs and mechanisms of persistence of *Vibrio cholerae*. Front. Microbiol. **4**, 375 (2013). 24379807 10.3389/fmicb.2013.00375 PMC3863721

[3] M. del Refugio Castañeda Chávez, V. P. Sedas, E. O. Borunda, F. L. Reynoso, Influence of water temperature and salinity on seasonal occurrences of *Vibrio cholerae* and enteric bacteria in oyster-producing areas of Veracruz, México. Mar. Pollut. Bull. **50**, 1641–1648 (2005). 16061261 10.1016/j.marpolbul.2005.06.036

[4] A. Huq, P. A. West, E. B. Small, M. I. Huq, R. R. Colwell, Influence of water temperature, salinity, and pH on survival and growth of toxigenic *Vibrio cholerae* serovar 01 associated with live copepods in laboratory microcosms. Appl. Environ. Microbiol. **48**, 420–424 (1984). 6486784 10.1128/aem.48.2.420-424.1984 PMC241529

[5] F. P. Rothenbacher, J. Zhu, Efficient responses to host and bacterial signals during *Vibrio cholerae* colonization. Gut Microbes **5**, 120–128 (2014). 24256715 10.4161/gmic.26944 PMC4049929

[6] M. Grognot, A. Mittal, M. Mah’moud, K. M. Taute, *Vibrio cholerae* motility in aquatic and mucus-mimicking environments. Appl. Environ. Microbiol. **87** (2021). 10.1128/AEM.01293-21 PMC8478464 34347522

[7] J. K. Teschler , Living in the matrix: Assembly and control of *Vibrio cholerae* biofilms. Nat. Rev. Microbiol. **13**, 255–268 (2015). 25895940 10.1038/nrmicro3433 PMC4437738

[8] S. M. Butler, A. Camilli, Going against the grain: Chemotaxis and infection in *Vibrio cholerae*. Nat. Rev. Microbiol. **3**, 611–620 (2005). 16012515 10.1038/nrmicro1207 PMC2799996

[9] X. Zhang , Amino acid-induced chemotaxis plays a key role in the adaptation of *Vibrio harveyi* from seawater to the muscle of the host fish. Microorganisms **12**, 1292 (2024). 39065061 10.3390/microorganisms12071292 PMC11278769

[10] J. Xu , The role of morphological adaptability in *Vibrio cholerae*’s motility. mBio **16**, e02469-24 (2025). 39611848 10.1128/mbio.02469-24 PMC11708025

[11] A. S. Utada , *Vibrio cholerae* use pili and flagella synergistically to effect motility switching and conditional surface attachment. Nat. Commun. **5**, 4913 (2014). 25234699 10.1038/ncomms5913 PMC4420032

[12] M. Gomelsky, W. D. Hoff, Light helps bacteria make important lifestyle decisions. Trends Microbiol. **19**, 441–448 (2011). 21664820 10.1016/j.tim.2011.05.002

[13] R. Macnab, D. E. Koshland, Bacterial motility and chemotaxis: Light-induced tumbling response and visualization of individual flagella. J. Mol. Biol. **84**, 399–406 (1974). 4618854 10.1016/0022-2836(74)90448-3

[14] S. Wright, B. Walia, J. S. Parkinson, S. Khan, Differential activation of *Escherichia coli* chemoreceptors by blue-light stimuli. J. Bacteriol. **188**, 3962–3971 (2006). 16707688 10.1128/JB.00149-06 PMC1482890

[15] M. Iwanaga , Characteristic of *Vibrio cholerae* O1 isolated in the Aja River. Kansenshogaku. Zasshi. **59**, 551–558 (1985). 3932541 10.11150/kansenshogakuzasshi1970.59.551

[16] K. A. McDonough, A. Rodriguez, The myriad roles of cyclic AMP in microbial pathogens: From signal to sword. Nat. Rev. Microbiol. **10**, 27–38 (2012). 10.1038/nrmicro2688 PMC3785115 22080930

[17] A. Kolb, S. Busby, H. Buc, S. Garges, S. Adhya, Transcriptional regulation by cAMP and its receptor protein. Annu. Rev. Biochem. **62**, 749–797 (1993). 8394684 10.1146/annurev.bi.62.070193.003533

[18] M. Stierl , Light modulation of cellular cAMP by a small bacterial photoactivated adenylyl cyclase, bPAC, of the soil bacterium *Beggiatoa*. J. Biol. Chem. **286**, 1181–1188 (2011). 21030594 10.1074/jbc.M110.185496 PMC3020725

[19] M. Efetova, M. Schwärzel, Photoactivatable adenylyl cyclases (PACs) as a tool to study cAMP signaling in vivo: An overview. Methods Mol. Biol. **1294**, 131–135 (2015). 25783882 10.1007/978-1-4939-2537-7_10

[20] Y. Nakasone, H. Murakami, S. Tokonami, T. Oda, M. Terazima, Time-resolved study on signaling pathway of photoactivated adenylate cyclase and its nonlinear optical response. J. Biol. Chem. **299**, 105285 (2023). 37742920 10.1016/j.jbc.2023.105285 PMC10634658

[21] C. C. Häse, B. Barquera, Role of sodium bioenergetics in *Vibrio cholerae*. Biochim. Biophys. Acta (BBA), Bioenergetics **1505**, 169–178 (2001). 11248198 10.1016/s0005-2728(00)00286-3

[22] N. Takekawa , Sodium-driven energy conversion for flagellar rotation of the earliest divergent hyperthermophilic bacterium. Sci. Rep. **5**, 12711 (2015). 26244427 10.1038/srep12711 PMC4525482

[23] M. Iseki , A blue-light-activated adenylyl cyclase mediates photoavoidance in *Euglena gracilis*. Nature **415**, 1047–1051 (2002). 11875575 10.1038/4151047a

[24] J.-Y. Tinevez , TrackMate: An open and extensible platform for single-particle tracking. Methods **115**, 80–90 (2017). 27713081 10.1016/j.ymeth.2016.09.016

[25] R. N. C. Buensuceso , Cyclic AMP-independent control of twitching motility in *Pseudomonas aeruginosa*. J. Bacteriol. **199**, e00188-17 (2017). 28583947 10.1128/JB.00188-17 PMC5527385

[26] L. Xu , A cyclic di-GMP–binding adaptor protein interacts with a chemotaxis methyltransferase to control flagellar motor switching. Sci. Signal. **9**, ra102 (2016). 27811183 10.1126/scisignal.aaf7584

[27] V. M. Suchanek , Chemotaxis and cyclic-di-GMP signalling control surface attachment of *Escherichia coli*. Mol. Microbiol. **113**, 728–739 (2020). 31793092 10.1111/mmi.14438

[28] J. Xu , Light dependent synthesis of a nucleotide second messenger controls the motility of a spirochete bacterium. Sci. Rep. **12**, 6825 (2022). 35474318 10.1038/s41598-022-10556-7 PMC9043183

[29] J. T. O. Kirk, Light and Photosynthesis in Aquatic Ecosystems (Cambridge University Press, 1994).

[30] Y. Wang , Tidal variability of phytoplankton distribution in the highly turbid Changjiang River Estuary: Mechanisms and implications. J. Geophys. Res. Oceans **128**, e2023JC020090 (2023).

[31] D.-P. Häder, New trends in photobiology. J. Photochem. Photobiol. B **1**, 385–414 (1988).

[32] D. H. McLachlan, C. Brownlee, A. R. Taylor, R. J. Geider, G. J. C. Underwood, Light-induced motile responses of the estuarine benthic diatoms *Navicula perminuta* and *Cylindrotheca closterium* (Bacillariophyceae)^1^. J. Phycol. **45**, 592–599 (2009). 27034035 10.1111/j.1529-8817.2009.00681.x

[33] Y. I. Posudin, N. P. Massjuk, G. G. Lilitskaya, “Terminology and the fundamentals of classification of light-induced behaviour in freely motile microorganisms” in *Photomovement of Dunaliella Teod*, U. Wrasmann, A. Wilke, Eds. (Vieweg+Teubner Verlag, 2010), pp. 13–22.

[34] G. Frangipane , Dynamic density shaping of photokinetic *E. coli*. eLife **7**, e36608 (2018). 30103856 10.7554/eLife.36608 PMC6092124

[35] K. K. Gosink, C. C. Häse, Requirements for conversion of the Na^+^-Driven flagellar motor of *Vibrio cholerae* to the H^+^-Driven motor of *Escherichia coli*. J. Bacteriol. **182**, 4234–4240 (2000). 10894732 10.1128/jb.182.15.4234-4240.2000 PMC101923

[36] P. Halang, T. Vorburger, J. Steuber, Serine 26 in the PomB subunit of the flagellar motor is essential for hypermotility of *Vibrio cholerae*. PLoS One **10**, e0123518 (2015). 25874792 10.1371/journal.pone.0123518 PMC4398553

[37] T.-S. Lin , Stator dynamics depending on Sodium concentration in Sodium-driven bacterial flagellar motors. Front. Microbiol. **12** (2021). 10.3389/fmicb.2021.765739 PMC8661058 34899649

[38] B. R. Boles, L. L. McCarter, Insertional inactivation of genes encoding components of the sodium-type flagellar motor and switch of *Vibrio parahaemolyticus*. J. Bacteriol. **182**, 1035–1045 (2000). 10648530 10.1128/jb.182.4.1035-1045.2000 PMC94380

[39] A. Banerjee , In vivo nitrosative stress-induced expression of a photolyase promotes *Vibrio cholerae* environmental blue light resistance. Mol. Microbiol. **123**, 295–304 (2025), 10.1111/mmi.15340. 39814688 PMC11976125

[40] C. Van der Henst , Molecular insights into *Vibrio cholerae*’s intra-amoebal host-pathogen interactions. Nat. Commun. **9**, 3460 (2018). 30150745 10.1038/s41467-018-05976-x PMC6110790

[41] T. R. Schleicher, S. V. Nyholm, Characterizing the host and symbiont proteomes in the association between the bobtail squid, *Euprymna scolopes*, and the bacterium, *Vibrio fischeri*. PLoS One **6**, e25649 (2011). 21998678 10.1371/journal.pone.0025649 PMC3187790

[42] T. Miyashiro, E. G. Ruby, Shedding light on bioluminescence regulation in *Vibrio fischeri*. Mol. Microbiol. **84**, 795–806 (2012). 22500943 10.1111/j.1365-2958.2012.08065.x PMC3359415

[43] B. L. Taylor, D. E. Koshland, Intrinsic and extrinsic light responses of *Salmonella typhimurium* and *Escherichia coli*. J. Bacteriol. **123**, 557–569 (1975). 1097417 10.1128/jb.123.2.557-569.1975 PMC235761

[44] S. Masuda, Light detection and signal transduction in the BLUF photoreceptors. Plant Cell Physiol. **54**, 171–179 (2013). 23243105 10.1093/pcp/pcs173

[45] M. Gomelsky, G. Klug, BLUF: A novel FAD-binding domain involved in sensory transduction in microorganisms. Trends Biochem. Sci. **27**, 497–500 (2002). 12368079 10.1016/s0968-0004(02)02181-3

[46] O. A. Soutourina, P. N. Bertin, Regulation cascade of flagellar expression in Gram-negative bacteria. FEMS Microbiol. Rev. **27**, 505–523 (2003). 14550943 10.1016/S0168-6445(03)00064-0

[47] L. L. McCarter, Dual flagellar systems enable motility under different circumstances. Microb. Physiol. **7**, 18–29 (2004). 10.1159/000077866 15170400

[48] R. Stocker, Marine microbes see a sea of gradients. Science **338**, 628–633 (2012). 23118182 10.1126/science.1208929

[49] J. M. Keegstra , Risk–reward trade-off during carbon starvation generates dichotomy in motility endurance among marine bacteria. Nat. Microbiol. **10**, 1393–1403 (2025). 40419768 10.1038/s41564-025-01997-7 PMC12137127

[50] M. Wölflingseder, S. Tutz, V. H. Fengler, S. Schild, J. Reidl, Regulatory interplay of RpoS and RssB controls motility and colonization in *Vibrio cholerae*. Int. J. Med. Microbiol. **312**, 151555 (2022). 35483107 10.1016/j.ijmm.2022.151555

[51] M. L. Tamplin, A. L. Gauzens, A. Huq, D. A. Sack, R. R. Colwell, Attachment of *Vibrio cholerae* serogroup O1 to zooplankton and phytoplankton of Bangladesh waters. Appl. Environ. Microbiol. **56**, 1977–1980 (1990). 2383016 10.1128/aem.56.6.1977-1980.1990 PMC184543

[52] R. R. Colwell, A. Huq, Environmental reservoir of *Vibrio cholerae* the causative agent of choleraa. Ann. N. Y. Acad. Sci. **740**, 44–54 (1994). 7840478 10.1111/j.1749-6632.1994.tb19852.x

[53] M. Emch, C. Feldacker, M. S. Islam, M. Ali, Seasonality of cholera from 1974 to 2005: A review of global patterns. Int. J. Health Geogr. **7**, 31 (2008). 18570659 10.1186/1476-072X-7-31 PMC2467415

[54] T. Baracchini , Seasonality in cholera dynamics: A rainfall-driven model explains the wide range of patterns in endemic areas. Adv. Water Resour. **108**, 357–366 (2017).

[55] R. R. Colwell, Global climate and infectious disease: The cholera paradigm. Science **274**, 2025–2031 (1996). 8953025 10.1126/science.274.5295.2025

[56] E. K. Lipp, A. Huq, R. R. Colwell, Effects of global climate on infectious disease: The cholera model. Clin. Microbiol. Rev. **15**, 757–770 (2002). 12364378 10.1128/CMR.15.4.757-770.2002 PMC126864

[57] L. M. Walker , A simple mechanism for integration of quorum sensing and cAMP signalling in *Vibrio cholerae*. eLife **12**, RP86699 (2023). 37410076 10.7554/eLife.86699 PMC10328515

[58] F. Taguchi, Y. Ichinose, Virulence factor regulator (Vfr) controls virulence-associated phenotypes in *Pseudomonas syringae* pv. *tabaci* 6605 by a quorum sensing-independent mechanism. Mol. Plant Pathol. **14**, 279–292 (2013). 23145783 10.1111/mpp.12003 PMC6638821

[59] S.-P. Kim, C.-M. Kim, S.-H. Shin, Cyclic AMP and cyclic AMP-receptor protein modulate the Autoinducer-2-mediated quorum sensing system in *Vibrio vulnificus*. Curr. Microbiol. **65**, 701–710 (2012). 22961036 10.1007/s00284-012-0218-0

[60] J. Xu, Raw data of “Light-activated cAMP signaling controls sodium-driven motility in Vibrio cholerae. Mendeley Data, V1, doi:10.17632/23s5y33dn9.1. Deposited 10 October 2025. PMC13079933 41955113

