# Supplementary material for: Light-activated cAMP signaling controls sodium-driven motility in Vibrio cholerae
Source: Proc Natl Acad Sci U S A. 2026 Apr 9;123(15):e2530860123. doi: 10.1073/pnas.2530860123 (PMC13079933; doi:10.1073/pnas.2530860123)
Supplement: Supplementary file 1 — Appendix 01 (PDF) [file pnas.2530860123.sapp.pdf]

PNAS

**Supporting Information for**
**Light-activated cAMP signaling controls sodium-driven motility in *Vibrio cholerae***

Jun Xu[a*], Shuichi Nakamura[b], Suzuna Tomoyose[a], Reika Shimabuku[a], Rintaro Tomioka[c] , Tetsu Yamashiro[a*]

**Corresponding authors:** Jun Xu, Tetsu Yamashiro
**Email:** xujunbac@cs.u-ryukyu.ac.jp, tyamashi@cs.u-ryukyu.ac.jp

**This PDF file includes:**

Figures S1 to S9
Supportive information S.I.1
Legends for Movies S1 to S8
Supplementary methods and materials
Supplementary References

**Other supporting materials for this manuscript include the following:**

Movies S1 to S8

**Supporting Information**

**Fig. S1.**

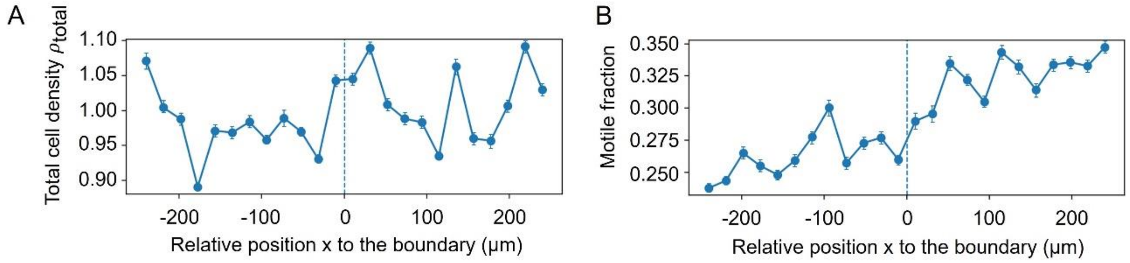


**Fig. S1. Spatial profiles of total cell density and motile fraction across the step gradient illumination boundary (in Fig. 2).** *(A)* Total cell density ($\boldsymbol{\rho}_{\mathbf{total}}$) as a function of position x relative to the step boundary. For each frame, all detected cells were binned along the x-axis to obtain a per-frame density profile, which was normalized to the frame-mean density and then averaged across frames. Values are shown as mean ± SEM across frames. *(B)* Motile fraction as a function of position x. Within each x-bin, motile fraction was calculated as $\boldsymbol{N}_{\mathbf{motile}}/\boldsymbol{N}_{\mathbf{total}}$ , where motile cells were defined using the same motility threshold applied in the tracking analyses. Values are shown as mean ± SEM across frames. The dashed vertical line indicates the boundary center ($\boldsymbol{x} = \mathbf{0}$); negative and positive x correspond to the dim and bright sides, respectively. Together, these profiles indicate that overall cell abundance is approximately uniform across the field of view, whereas the fraction of motile cells increases toward the bright region, which likely contributes to the apparent enrichment of motile trajectories in bright areas (Fig. 2B) alongside the modest “brightward” bias quantified in Fig. 2D,E.

**Fig. S2.**

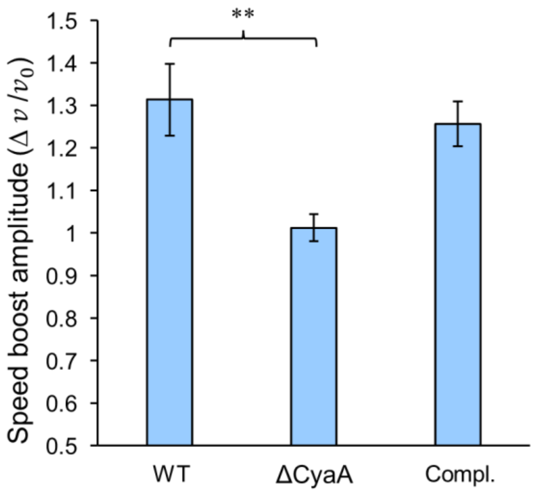


**Fig. S2. Fold Change of Light-Induced Motility Enhancement in *V. cholerae*.** Swimming speeds of wild-type (WT), cyaA deletion mutant (ΔcyaA), and complemented (Compl.) strains under bright light were normalized to their respective baseline motility under dim light, set as 1 for each strain. WT and complemented strains exhibited a significant fold increase in swimming speed upon light exposure, while the ΔcyaA strain showed minimal change. Data represent mean ± SD from three independent experiments. Statistical significance was determined by one-way ANOVA with Tukey's post hoc test; $^{**}p < 0.01$.

**Fig. S3.**

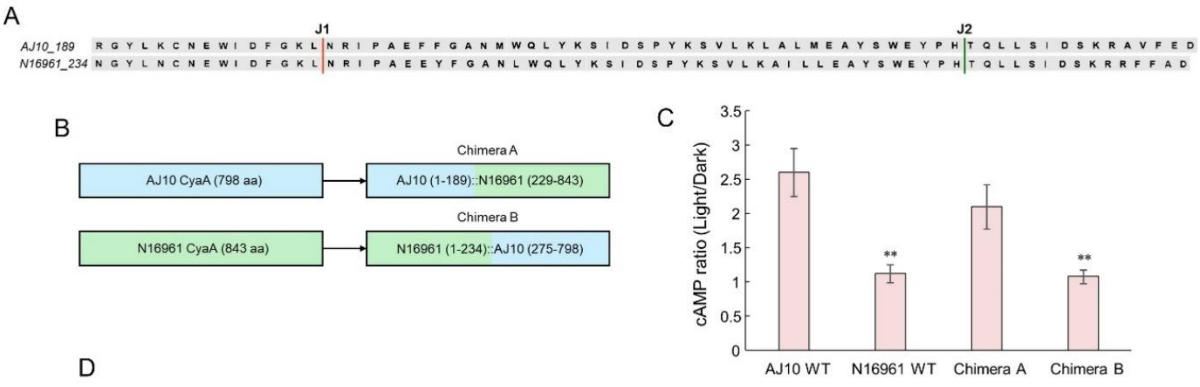


| Primer name | Sequence (5' → 3') | Purpose/Notes |
|---|---|---|
| Vec-F | AGACCATTCGGATGCAGACTTCATGCTAGCTAGGATCCGTCGACAA | Vector PCR (left) – rc (AJ10 5' overlap) + vector bind |
| Vec-R | TTAACGGTAAGCCTAGACGATCAACGGTTGACGATCTAGGACCTGA | Vector PCR (right) – rc (N16961 3' overlap) + vector bind |
| AJ10-N-F | ATGAAGTCTGCATCCGAATGGTCTATGAAGTCTGCATCCGAATGGT | Fragment A 5' → vector (AJ10 start + vector overlap) |
| AJ10-N-R | TGAAGTTGCTGAGTTGACGAGTTCGGTACGATCAGGTCGAACTG | Fragment A 3' → Fragment B (J1 overlap: N16961) |
| N16961-core-F | TGAAGTTGCTGAGTTGACGAGTTCGTTGACGAGTTCCGATGACC | Fragment B 5' → Fragment A (J1 overlap: N16961) |
| N16961-core-R | TTGATCGTCTAGGCTTACCGTTAATTAACGGTAAGCCTAGACGATC | Fragment B 3' → vector (N16961 stop + vector overlap) |
| VecB-F | TAATCCGGAGTCGTTAACCGACATGCTAGCTAGGATCCGTCGACAA | Vector PCR (left) – rc (N16961 5' overlap) + vector bind |
| VecB-R | TTAAGGCCATCGATACGGTTCAGCCGGTTGACGATCTAGGACCTGA | Vector PCR (right) - rc (AJ10 3' overlap) + vector bind |
| N16961-N-F | ATGTCGGTTAACGACTCCGGATTAATGTCGGTTAACGACTCCGGAT | Fragment A 5' → vector (N16961 start + vector overlap) |
| N16961-N-R | CCTGATCGTACCACCGTCTGGAATTCAGCAACTTCAGGTCCGAT | Fragment A 3' → Fragment B (J1 overlap: AJ10) |
| AJ10-core-F | CCTGATCGTACCACCGTCTGGAATACCGTCTGGAATGACCGTTA | Fragment B 5' → Fragment A (J1 overlap: AJ10) |
| AJ10-core-R | GCTGAACCGTATCGATGGCCTTAATTAAGGCCATCGATACGGTTCA | Fragment B 3' → vector (AJ10 stop + vector overlap) |
| AJ10-FL-F | ATGAAGTCTGCATCCGAATGGTCTATGAAGTCTGCATCCGAATGGT | AJ10 full-length insert 5' → vector |
| AJ10-FL-R | GCTGAACCGTATCGATGGCCTTAATTAAGGCCATCGATACGGTTCA | AJ10 full-length insert 3' → vector |
| N16961-FL-F | ATGTCGGTTAACGACTCCGGATTAATGTCGGTTAACGACTCCGGAT | N16961 full-length insert 5' → vector |
| N16961-FL-R | TTGATCGTCTAGGCTTACCGTTAATTAACGGTAAGCCTAGACGATC | N16961 full-length insert 3' → vector |

**Fig. S3. Domain-swap analysis of CyaA between *V. cholerae* strains AJ10 and N16961.** *(A)* Sequence alignment of CyaA proteins from AJ10 and N16961 highlighting the junction points (J1 and J2, red and green lines, respectively) selected for constructing chimeras. Conserved residues are shaded in grey. *(B)* Schematic of domain-swapped constructs. Chimera A consists of the N-terminal region of AJ10 (residues 1–189) fused to the C-terminal region of N16961 (residues 229–843), while Chimera B consists of the N-terminal region of N16961 (residues 1–234) fused to the C-terminal region of AJ10 (residues 275–798). *(C)* Light-to-dark cAMP ratios measured in *E. coli* MG1655 Δ*cyaA* Δ*cpdA* expressing the indicated constructs (AJ10 WT, N16961 WT, Chimera A, Chimera B), so that cAMP originates solely from plasmid-encoded CyaA variants (conditions as in Methods). AJ10 WT showed a strong light-induced cAMP increase, N16961 WT and Chimera B showed little response, and Chimera A retained partial responsiveness with reduced amplitude relative to AJ10 WT. *(D)* Primers used for construction of chimeric and full-length cyaA genes.

**Fig. S4.**

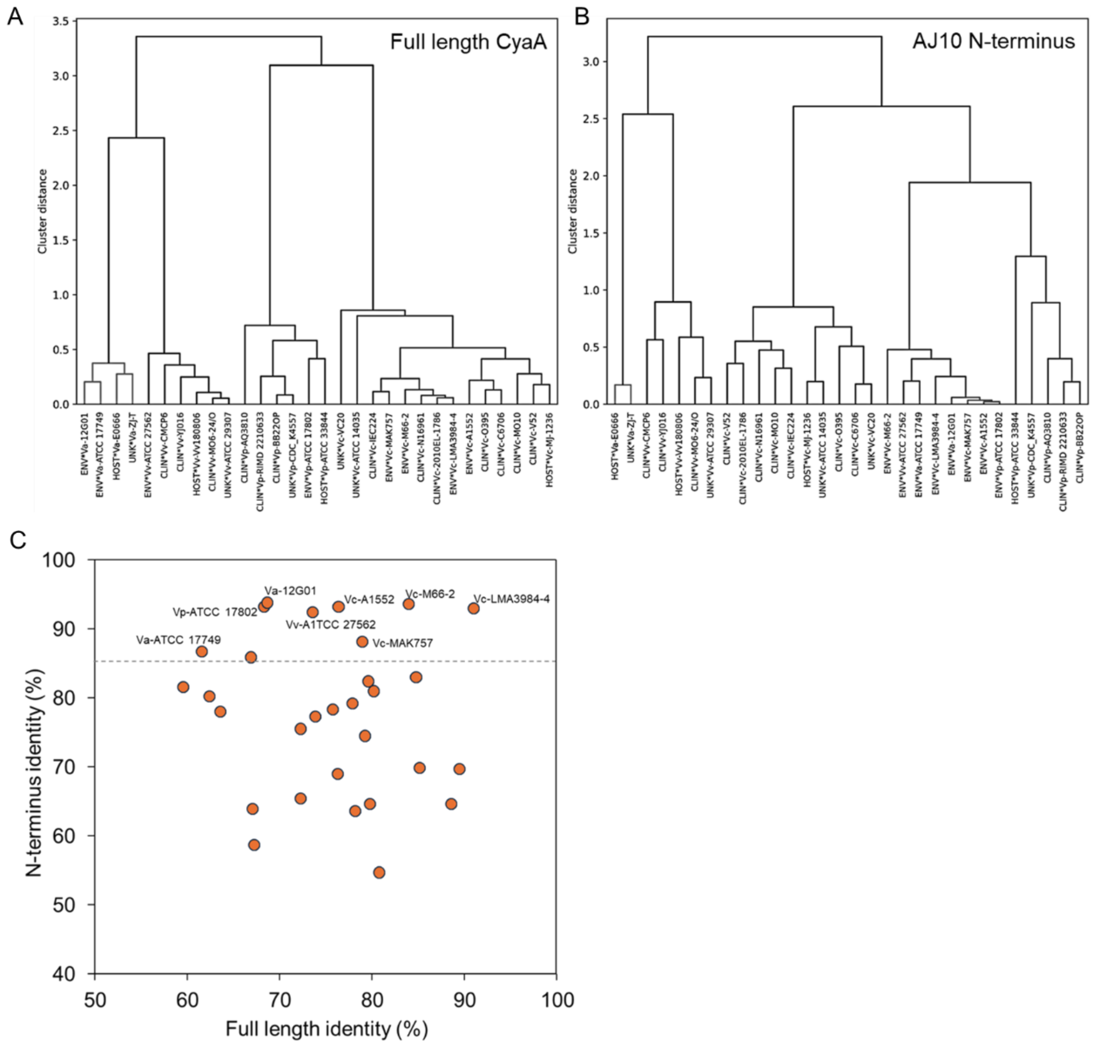


**Fig. S4. Comparative sequence similarity analysis of CyaA across representative *Vibrio* strains.** Protein sequences of CyaA homologs were retrieved from genome annotations for the Vibrio strains. Pairwise amino-acid identities were computed by aligning each sequence to AJ10 CyaA (full length or N-terminal segment, as indicated) and calculating percent identity over the aligned region. For phylogeny dendrogram, a distance matrix was generated from pairwise similarities (distance = 1 - normalized identity) and clustered by agglomerative hierarchical clustering (average linkage) to visualize relationships among strains. *(A)* Hierarchical clustering based on pairwise similarity of the full-length CyaA (cyaA) protein from the indicated strains. Prefix labels indicate isolate/source category and species: CLIN (clinical), ENV (environmental), HOST (host-associated), and UNK (unknown); Vc (*V. cholerae*), Vp (*V. parahaemolyticus*), Vv (*V. Vulnificus*), Va (*V. Alginolyticus*). *(B)* Hierarchical clustering based on pairwise similarity of the AJ10 CyaA N-terminal segment (the region used for AJ10-like classification). *(C)* Scatter plot showing full-length identity (%) versus N-terminal identity (%) of each strain relative to AJ10 CyaA. The dashed horizontal line indicates the example cutoff used to define an “AJ10-like” N-terminus (≥85% identity over the N-terminal segment).

**Fig. S5.**

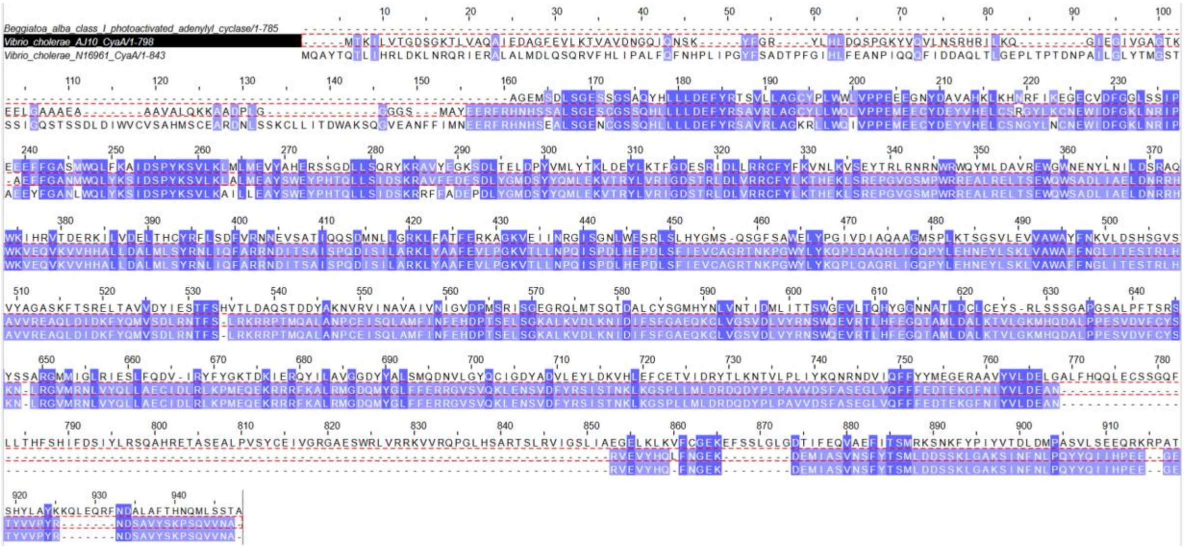


**Fig. S5. Alignment of AJ10 CyaA with Photoactivated Cyclase.** To investigate the molecular basis of light-induced cAMP signaling in *V. cholerae* AJ10, we performed BLASTp analysis of its CyaA protein sequence against a range of known photoactivated adenylyl cyclases (PACs), including those from *Cyanobacteria*, *Oscillatoria*, *Turneriella parva*, and *Beggiatoa alba*. Among these, the PAC from *Beggiatoa* exhibited the highest similarity, with 33% identity and 52% similarity over a 613 amino acid alignment, corresponding to the conserved cyclase homology domain (CHD). This strong structural homology confirms that AJ10 CyaA retains a functional cAMP-synthesizing core. While canonical light-sensing motifs such as BLUF or LOV domains were not detected, the conservation with known PACs raises the possibility that AJ10 CyaA may be regulated by alternative or strain-specific light-responsive mechanisms. Furthermore, direct comparison of AJ10 CyaA to the homologous sequence in the non-photoresponsive *V. cholerae* strain N16961 revealed overall high similarity but with notable sequence divergence near the N-terminal region. These differences may underline the strain-specific gain-of-function adaptation observed in AJ10, enabling light-dependent regulation of cAMP production and motility. Together, these findings support our proposed model that the AJ10 variant of CyaA plays a central role in linking environmental light cues to behavioral responses in *V. cholerae*.

**Fig. S6.**

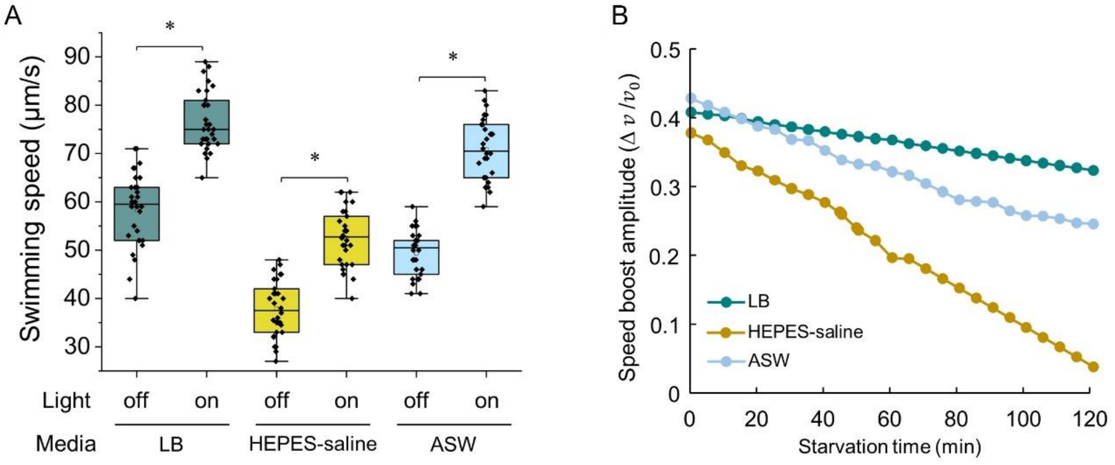


**Fig. S6. Effect of nutrient availability and starvation on light-induced motility enhancement.** *(A)* Swimming speeds of *V. cholerae* cells measured under light-off and light-on conditions in different media. In nutrient-rich LB, illumination significantly increased swimming speed. Cells suspended in HEPES-saline buffer (nutrient-free, isotonic) displayed lower baseline speeds but still showed a modest light-induced boost. In artificial seawater (ASW), which mimics natural aquatic conditions, cells maintained an intermediate baseline speed and also exhibited a significant light response. Each dot represents an individual cell; boxes indicate interquartile range with median, whiskers show 10th-90th percentiles. $P < 0.05$ (paired *t*-test). *(B)* Persistence of the motility boost under starvation. Cells pre-grown in LB were washed and resuspended in the indicated media (LB, HEPES-saline, or ASW) and incubated without added nutrients. The amplitude of the light-induced speed boost ($\Delta v/v_0$) was calculated relative to the dark speed baseline and plotted as a function of starvation time. The response gradually declined in all conditions but remained most stable in LB, followed by ASW, and decreased most rapidly in HEPES-saline. These results indicate that the photokinetic effect depends on cellular energy status and is sustained better in nutrient-rich or environmentally relevant media than under nutrient-depleted saline.

**Fig. S7.**

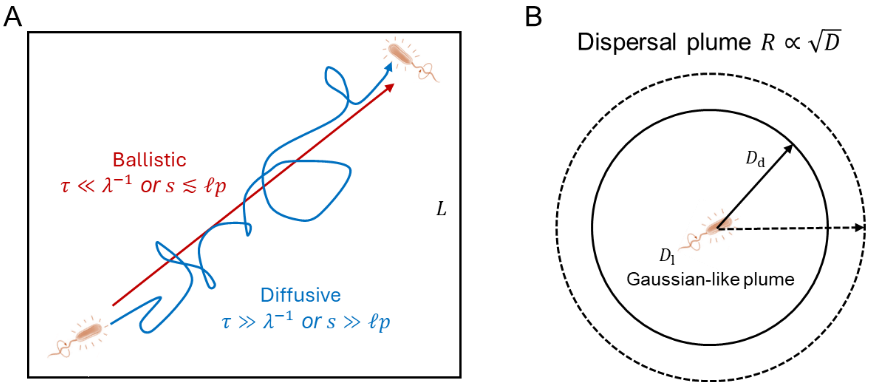


**Fig.S7 Scaling estimates for how a light-induced speed increase alters encounter and dispersal.** (A) We denote dim (low light) and light conditions by subscripts $d$ and $\ell$, respectively: mean speed $v_{\mathrm{d}}, v_{\mathrm{l}}$, reorientation rate $\lambda_{\mathrm{d}}, \lambda_{\mathrm{l}}$, and effective diffusion coefficient $D_{\mathrm{d}}, D_{\mathrm{l}}$. In a persistent random walk with mean speed $v$ and reorientation rate $\lambda$, the persistence length is $\ell_{\mathrm{P}} = v/\lambda$. At short times/distances (ballistic regime; $\tau \ll \lambda^{-1}$or $s \lesssim \ell_{\mathrm{P}}$), encounter rate scales linearly with speed ($k \propto v$). At long times/distances (diffusive regime; $\tau \gg \lambda^{-1}$or $s \gg \ell_{\mathrm{P}}$), the effective translational diffusion coefficient scales as $D \propto v^2/\lambda$, and diffusion-limited encounter rates scale as $k \propto D$. Therefore, a fractional speed increase $v_{\mathrm{l}} = (1+\varepsilon)v_{\mathrm{d}}$ yields $k_{\mathrm{l}}/k_{\mathrm{d}} = 1+\varepsilon$ in the ballistic limit and $k_{\mathrm{l}}/k_{\mathrm{d}} = (1+\varepsilon)^2$ in the diffusive limit (here $\varepsilon = 0.30$, giving +30% and +69%, respectively). (B) Dispersal scaling: for a Gaussian-like spreading cloud, a characteristic plume radius grows as $R(t) \propto \sqrt{Dt}$; thus $R_{\mathrm{l}}(t)/R_{\mathrm{d}}(t) = \sqrt{D_{\mathrm{l}}/D_{\mathrm{d}}} = (1+\varepsilon)$, i.e., ~30% larger radius at fixed time for $\varepsilon = 0.30$. Detailed derivations and assumptions are provided in S.I.1.

**S.I.1. Mathematical modeling: impact of a speed increase on encounter probability and dispersal.**

**Empirical basis:**

Over the analyzed time window, illumination increases the mean swimming speed of *V. cholerae* by ~30%, while turning rate $\lambda$ change only modestly (Fig. *2C-D*).

**Model and assumptions:**

We approximate cell motion as a persistent random walk with (i) mean speed $v$ and (ii) reorientation rate $\lambda$(turns $\mathrm{s}^{-1}$). The persistence length is:

$$\ell_{\mathrm{P}} = \frac{v}{\lambda}. \qquad [1]$$

Illumination is modeled as a speed-only perturbation:

$$v_{\mathrm{l}} = (1+\varepsilon)v_{\mathrm{d}}, \varepsilon = 0.30, \qquad [2]$$

with $\lambda$ unchanged to first order over the same time window.

We consider two limiting regimes defined either by observation time $\tau$or observation length scale $s$:

$$\textbf{Ballistic: } \tau \ll \lambda^{-1}(\text{or } s \lesssim \ell_{\mathrm{P}}).$$
$$\textbf{Diffusive: } \tau \gg \lambda^{-1}(\text{or } s \gg \ell_{\mathrm{P}}). \qquad [3]$$

**Effective diffusion coefficient:**

At long times, the motion coarse-grains to diffusion with:

$$D = \kappa_{\mathrm{d}} \frac{v^2}{\lambda}, \qquad [4]$$

Here $K_{\mathrm{d}}$ is a dimensionless prefactor that depends on the dimensionality (2D vs 3D) and on how reorientation is modeled (e.g., Poisson 'tumble' events that reset the heading vs continuous rotational diffusion) (1, 2). Importantly, $K_{\mathrm{d}}$ cancels in the ratio $D_{\mathrm{l}}/D_{\mathbf{d}}$, so its exact value is not required for our scaling comparison.

$$D_{\mathrm{l}} = K_{\mathrm{d}} \frac{v_{\mathrm{l}}^2}{\lambda_{\mathrm{l}}}, \quad D_{\mathrm{d}} = K_{\mathrm{d}} \frac{v_{\mathrm{d}}^2}{\lambda_{\mathrm{d}}} \qquad [5]$$

Over the analyzed time window, illumination increases speed while the reorientation rate changes only modestly (Fig. *2D*), so we take $\lambda_{\mathrm{l}} \approx \lambda_{\mathrm{d}}$ to first order. Therefore,

$$\frac{D_{\mathrm{l}}}{D_{\mathrm{d}}} \approx \left(\frac{v_{\mathrm{l}}}{v_{\mathrm{d}}}\right)^2 = (1+\varepsilon)^2 \qquad [6]$$

**Result 1: Encounter rate scaling**

For sparse targets, encounter rates scale differently in the two regimes:

**Ballistic (transport-limited):** $k_{\mathrm{bal}} \propto v$, therefore

$$\frac{k_{\mathrm{bal,l}}}{k_{\mathrm{bal,d}}} = \frac{v_{\mathrm{l}}}{v_{\mathrm{d}}} = 1 + \varepsilon = 1.30. \qquad [7]$$

**Diffusive (diffusion-limited):** $k_{\mathrm{diff}} \propto D$, therefore

$$\frac{k_{\mathrm{diff,l}}}{k_{\mathrm{diff,d}}} = \frac{D_{\mathrm{l}}}{D_{\mathrm{d}}} = (1+\varepsilon)^2 = 1.69. \qquad [8]$$

**Result 2: Traversal time across a layer of thickness $L$**

**Ballistic crossing.** In the ballistic regime, the characteristic traversal time across a layer of thickness $L$ scales as

$$t_{\mathrm{bal}} \sim \frac{L}{v}. \qquad [9]$$

With illumination modeled as a speed increase $v_{\mathrm{l}} = (1+\varepsilon)v_{\mathrm{d}}$, the ratio of traversal times is

$$\frac{t_{\mathrm{bal,l}}}{t_{\mathrm{bal,d}}} = \frac{v_{\mathrm{d}}}{v_{\mathrm{l}}} = \frac{1}{1+\varepsilon}. \qquad [10]$$

For $\varepsilon = 0.30$, this gives $t_{\mathrm{bal,l}}/t_{\mathrm{bal,d}} = 1/1.30 = 0.769$, i.e., ~23% faster traversal.

**Diffusive crossing.** In the diffusive regime, the characteristic traversal time scales as

$$t_{\mathrm{diff}} \sim \frac{L^2}{2D}. \qquad [11]$$

Using $D_{\mathrm{l}}/D_{\mathrm{d}} = (1+\varepsilon)^2$, the ratio of traversal times becomes

$$\frac{t_{\mathrm{diff,l}}}{t_{\mathrm{diff,\mathbf{d}}}} = \frac{D_{\mathrm{d}}}{D_{\mathrm{l}}} = \frac{1}{(1+\varepsilon)^2}. \qquad [12]$$

For $\varepsilon = 0.30$, this gives $t_{\mathrm{diff,l}}/t_{\mathrm{diff,\mathbf{d}}} = 1/1.30^2 = 0.592$, i.e., ~41% faster traversal.

**Result 3: Dispersal plume expansion**

Approximating the spreading population as Gaussian-like at long times, a characteristic radius scales as:

$$R(t) \sim \sqrt{2dDt}, \qquad [13]$$

so, at fixed $t$,

$$\frac{R_{\mathrm{l}}(t)}{R_{\mathrm{d}}(t)} = \sqrt{\frac{D_{\mathrm{l}}}{D_{\mathrm{d}}}} = 1 + \varepsilon = 1.30. \qquad [14]$$

**Interpretation**

A ~30% increase in speed produces a +30% (ballistic) to +69% (diffusive) increase in encounter rates and ~23-40% reductions in traversal times, depending on whether motion is effectively ballistic or diffusive over the relevant scale.

**Fig. S8.**

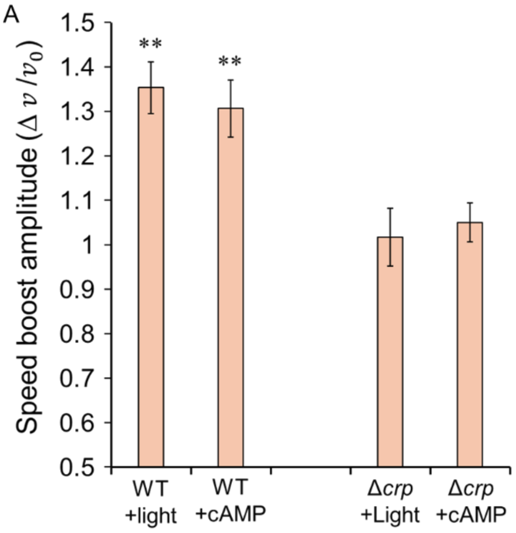


B

| Primer name | Sequence (5' → 3') | Purpose/Notes |
|---|---|---|
| crp-A | GATGAGCTCCGGTA GAAGTGGTCTTAGCG | Upstream Fwd *(SacI site)* |
| crp-B | TTCATTAGTACCAGTA CCTTCATCAGCTTTTC | Upstream Rev *(Overhang)* |
| crp-C | GAAAAGCTGATGAAG GTACTGGTACTAATGAA | Downstream Fwd (Overhang) |
| crp-D | GATTCTAGACCGCTGAA CCAAGATTAGCC | Downstream Rev (*XbaI* site) |
| crp-ext-F | GCTTGTCGAGCGTTAT GAGC | External Forward |
| crp-ext-R | CCAGTTGCTCGATACC TTGC | External Reverse |

**Fig. S8. CRP is required for the light- and cAMP-induced swimming speed boost.** (A) Speed boost amplitude is shown as the fold-change in mean swimming speed relative to the dark baseline, $\Delta v/v_0$, where $v_0$ is the mean swimming speed measured in the dim. WT cells exhibited a significant speed increase upon visible light exposure and upon addition of exogenous cAMP, whereas the Δ*crp* mutant showed no detectable increase under either condition. Bars represent mean ± SEM from 3 independent biological replicates. Statistical significance was relative to the corresponding dark baseline (**$P < 0.01$). (B) Primers used for construction of the Δ*crp* mutant.

**Fig. S9.**

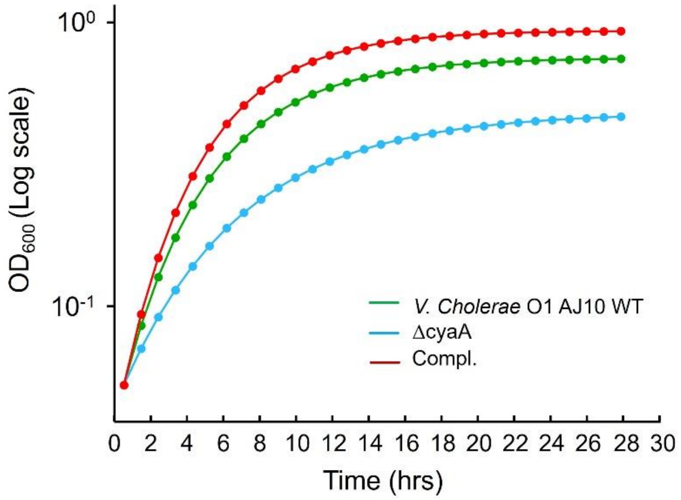


**Fig. S9. Growth of *V. cholerae* O1 AJ10 wild-type (WT), Δ*cyaA*, and complemented (Compl.).** strains in LB at 37 °C with shaking (200 rpm). Optical density ($OD_{600}$) was measured hourly and plotted on a logarithmic scale to highlight exponential growth. The Δ*cyaA* strain showed a slightly slower exponential phase but reached a similar stationary-phase density as WT and Compl.. This indicates the differences in baseline motility are not simply a result of poor cell fitness or population density differences.

## Legends for Movies S1 to S8

Movie S1 (separate file). Photokinesis in *V. cholerae* (400–700 nm).
Movie S2 (separate file). *V. cholerae* cells swimming in blue light (430–470 nm).
Movie S3 (separate file). *V. cholerae* cells swimming in green light (520–570 nm).
Movie S4 (separate file). *V. cholerae* cells swimming in red light (620–680 nm).
Movie S5 (separate file). *V. cholerae* cells swimming across light gradient.
Movie S6 (separate file). Swimming wide-type *V. cholerae* AJ10 cells upon light exposure.
Movie S7 (separate file). Swimming Δ*CyaA* cells upon light exposure.
Movie S8 (separate file). Swimming Δ*CyaA* cells with addition of cAMP in the dim.

## Supplementary Materials and Methods:

### Bacterial strains, culture medium, and reagents

The *Vibrio cholerae* O1 AJ10 strain used in this study is part of the AJ strain set (AJ1–AJ12) originally isolated from the Aja River (Naha–Urasoe area), Okinawa, Japan (3). The original report identified these isolates as *V. cholerae* O1, biotype El Tor, serotype Inaba, and described them as weakly pathogenic based on rabbit ileal loop assays, low intestinal adhesion, and minimal detectable cholera toxin production. The wild-type strain and genetically modified mutants were maintained in Luria-Bertani (LB) broth (1% tryptone, 0.5% yeast extract, 1% NaCl) at 37°C with shaking at 200 rpm. For solid medium, LB agar plates were supplemented with 1.5% agar. When necessary, antibiotics ampicillin (100 µg/mL) were added. HEPES–saline: 20 mM HEPES (pH 7.5), 120 mM NaCl, 5 mM KCl, 1 mM $MgCl_2$, 0.1 mM $CaCl_2$ (0.22 µm-filtered), and ASW (artificial seawater)+HEPES: commercial sea-salt (Sigma Aldrich, USA) mix 17.5 g $L^{-1}$ + 10–20 mM HEPES, pH 7.5 (0.22 µm-filtered) were also used to create starvation. Reagents for molecular biology experiments, including Taq polymerase, restriction enzymes, and T4 DNA ligase, were purchased from New England Biolabs (NEB, USA). The cAMP ELISA kit was obtained from Enzo Life Sciences (NY, USA). Membrane permeable cAMP (8-bromo cAMP) was purchased from Fujifilm (Tokyo, Japan). Membrane potential-sensitive dye $DiSC^3(5)$, Sodium-sensitive fluorescent dye SBFI-AM, were acquired from Thermo Fisher Scientific (USA) for membrane potential and intracellular sodium measurements.

### Microscopy and illumination setup

Cell suspensions (10 µL) were placed on a glass slide chamber and sealed with 0.09 mm double-sided tape and a 22 × 22 mm coverslip using Vaseline at the edges to minimize drift. Observations were performed using a darkfield microscope (Eclipse Ci-L Plus, Nikon, Japan) equipped with a 40× phase-contrast objective (NA 0.65). Illumination was from a phosphor-converted white LED epi source (Nikon Ci-S LED module).The SPD (spectral power distribution) measured at the specimen plane with a fiber-coupled array spectrometer (Ocean Optics USB2000+, 1 nm res., 50 ms integ.) showed a blue pump peak at 452 nm (FWHM 21 nm) and a broad phosphor band centered at 555 nm (FWHM 112 nm; spanning ~490–690 nm). Band-integrated photon fractions (mean of 3 measurements) were: <420 nm: 1.3%, 420–480 nm: 31%, 480–520 nm: 18%, 520–620 nm: 37%, >620–700 nm: 11%, >700 nm: 0.7%. Total out-of-band power (<420 nm and >700 nm) contributed ~2% of photons. Band-pass filters were also used in the experiments (430–470 nm, FWHM 46.7 nm, Tavg > 93%; 520–

570 nm, FWHM 55.3 nm, Tavg > 90%; or 620–680 nm, FWHM 60 nm, Tavg >95%; Asahi Spectra, Japan). Light intensity at the sample plane was measured using a photometer (LI-250A, LI-COR, USA) and expressed as photosynthetic photon flux density (PPFD; μmol photons m$^{-2}$ s$^{-1}$).
To test for directional motility responses, a step gradient light was generated by covering half of the LED light path with a translucent plastic film (Milky White Diffuser, As One Corp, Japan) (Fig. 2 *A*). This produced adjacent bright and dim regions across the same field of view, verified with the photometer. The bright side was measured at ~150 μmol m$^{-2}$ s$^{-1}$ PPFD, while the dim side remained below 10 μmol m$^{-2}$ s$^{-1}$. These PPFD values are within reported ranges for shallow/turbid coastal waters (4, 5).

**Video acquisition and trajectory analysis**

Movies were acquired at 60 frames per second (fps) using CMOS cameras (WAT-07U2, Watec, Japan; DFK-33UX273, The Imaging Source, ROC) mounted on a microscope equipped with a 40× phase-contrast objective (NA 0.65). Exposure time was 10 ms per frame. Each recording lasted 10–20 s and covered a field of view of 150 × 150 μm. Cell trajectories were tracked using the ImageJ TrackMate (NIH, USA) (6, 7), and identical acquisition and tracking procedures were applied across all conditions.

To ensure robust tracking, trajectories were included if cells remained in focus for ≥30 consecutive frames (0.5 s). Stage drift was corrected using fixed fiducial marks (debris adhered to the glass) as references; any global shift was estimated and subtracted from all trajectories. Instantaneous speed was computed as frame-to-frame displacement divided by the frame interval $(v(t) = \Delta r/\Delta t)$, and mean swimming speed was calculated by averaging $v(t)$ across the trajectory (or across each continuous motile segment, see below). Brief pauses (<10 consecutive frames with negligible displacement) were retained; longer immobile intervals (≥10 frames) were treated as a break and the track was split, with only segments meeting the minimum length criterion retained for speed analysis. For each biological replicate, ≥100 trajectories per region were analyzed.

For motile fraction, to avoid bias associated with net-displacement criteria, motile cells were defined using a speed-based threshold applied per trajectory: a trajectory was classified as motile if its mean speed exceeded $v_{\mathrm{th}}$= 5 μm/s. Cells below threshold were classified as non-motile and were included when calculating motile fraction (motile trajectories / total trajectories per field of view). We verified that the main conclusions were not sensitive to reasonable variation in $v_{\mathrm{th}}$.
Stage drift was corrected using fixed fiducial marks (debris adhered to the glass) as references, any global displacement was estimated from fiduciary motion and subtracted from all trajectories. For each condition, at least 100 trajectories per replicate were analyzed across three independent cultures, yielding >300 trajectories per condition in total. MATLAB analysis scripts are available in the deposited data.

**Boundary-crossing flux and drift velocity analysis in step gradient light**

A step-like illumination boundary was constructed by partially masking the illumination path using a translucent cover (Fig. 2 *A*). Time-lapse movies of swimming cells were analyzed by ImageJ TrackMate (7). Each trajectory consisted of time-ordered positions $(x(t), y(t))$ sampled at frame interval $\Delta t$. To quantify directional migration across the illumination step, we measured the flux of trajectories crossing the step boundary (8). A boundary zone of width 20 μm centered on the step edge was defined as $-10 \le x \le$

$+10$ μm (with $x = 0$ at the boundary center) (Fig. 2 *B*). Crossings were scored when a trajectory moved from one side of the boundary zone to the other without leaving the field of view during the transition. Specifically, a dim→bright crossing was counted if a trajectory moved from $x < -10$ μm to $x > +10$ μm, and a bright→dim crossing was counted if it moved from $x > +10$ μm to $x < -10$ μm.

For each movie, the crossing fluxes were computed as

$$J_{\mathrm{dim \to bright}} = \frac{N_{\mathrm{dim \to bright}}}{T}, \qquad J_{\mathrm{bright \to dim}} = \frac{N_{\mathrm{bright \to dim}}}{T},$$

where $N$ is the number of crossing events and $T$ is the movie duration. To control for differences in local swimmer abundance near the boundary, fluxes were normalized by the mean number of motile cells observed within the boundary zone during the movie, yielding a normalized crossing rate (crossings·cell$^{-1}$·s$^{-1}$). The flux directionality was presented as the ratio:

$$R_{\mathrm{J}} = \frac{J_{\mathrm{dim \to bright}}}{J_{\mathrm{bright \to dim}}}.$$

The value $R_{\mathrm{J}} = 1$ indicates symmetric boundary flux (non-directional migration), whereas $R_{\mathrm{J}} > 1$ indicates net migration toward the bright region, vice versa.

To quantify net drift along the bright-dim axis, instantaneous velocities were calculated from successive positions by finite differences:

$$v_{\mathrm{x}}(t_{\mathrm{i}}) = \frac{x(t_{\mathrm{i+1}}) - x(t_{\mathrm{i}})}{\Delta t}, \qquad v_{\mathrm{y}}(t_{\mathrm{i}}) = \frac{y(t_{\mathrm{i+1}}) - y(t_{\mathrm{i}})}{\Delta t}.$$

The projected velocity $v_{\mathrm{x}}$ (μm/s) indicates the instant component of motion along the x-axis. Positive $v_{\mathrm{x}}$ indicates motion toward the bright region, whereas negative $v_{\mathrm{x}}$ indicates motion toward the dim region. The no-drift reference is $v_{\mathrm{x}} = 0$. values from all included trajectories were pooled to construct the projected-velocity distribution.

**Soft agar motility assay**

Soft agar plates contained LB supplemented with 0.3% (w/v) agar (Difco). For testing in nutrient-limited conditions, the same agar concentration was prepared in ASW as indicated. A 1 μL drop of normalized culture was spotted onto the plate surface. Plates were incubated at 30 °C under either light-proof condition (ambient light <0.1 μmol m$^{-2}$ s$^{-1}$) or bright illumination (200 μmol m$^{-2}$ s$^{-1}$ white LED). Illumination was applied from the top with a uniform fiber-coupled LED lamp (Cold Spot, NPI, Japan) positioned 20 cm above the plates.

**Intracellular cAMP Measurement**

*V. cholerae* cells were grown in LB broth at 37°C with shaking to mid-log phase ($OD_{600}$ ≈ 0.5). Cells were then divided into groups and exposed to white light at varying illuminance levels for a fixed duration of 60s. Immediately following light exposure, 1 mL of each culture was rapidly mixed with 1 mL of ice-cold 100% ethanol to quench cellular activity and preserve intracellular cAMP. The mixtures were centrifuged at 10,000 × g for 10 minutes at 4°C, and the supernatants were collected. Ethanol was evaporated using a SpeedVac concentrator (Thermo Fisher Scientific), and the dried residues were resuspended in 0.1 M HCl. Intracellular cAMP concentrations were quantified using a competitive cAMP ELISA kit (Enzo Life Sciences, USA) according to the manufacturer's instructions. Standards and samples were added to a 96-well plate pre-coated with cAMP-specific antibodies. After sequential addition of enzyme conjugate and substrate solution, absorbance was measured at 405 nm using a

microplate reader. Final concentrations were determined by interpolating from a standard curve generated with known cAMP standards. Each condition was assayed in duplicate technical replicates, with three independent replicates.

### Construction of *cyaA* Knockout Mutants

*Vibrio cholerae* O1 AJ10 was cultured in LB broth at 37°C with shaking (200 rpm) to mid-log phase (OD600 ≈ 0.5), and genomic DNA was extracted using phenol-chloroform. Approximately 500 bp regions upstream and downstream of the *cyaA* gene (GenBank: NZ_CP043554.1) (9–11) were PCR-amplified using Phusion High-Fidelity DNA Polymerase (New England Biolabs, USA) and the following primers:
Up-F 5′-GGAATTCCGGTACGTGATGATGCTGA-3′
Up-R 5′-GCTGCAGCCAGTGTGGTTGAGGTTGATG-3′
Down-F 5′-GGAATTCCGTTGAGGCGTTTGAGTTGGA-3′
Down-R 5′-GCTGCAGCTGACGGTGATGTTGAGAGGC-3′
PCR conditions followed manufacturer instructions (Thermo Fisher Scientific), and amplicons were purified and digested with *EcoRI* and *PstI* (Takara Bio Inc, Japan). The fragments were gel-purified and ligated into the suicide vector pCVD442 (Addgene #11074) (10, 12) using T4 DNA ligase (New England Biolabs, USA). The ligation was transformed into *E. coli* DH5α, selected on LB-ampicillin (100 µg/mL), and verified by restriction digestion.
The validated plasmid was electroporated into electrocompetent *V. cholerae* AJ10 (2.5 kV, 200 Ω, 25 µF), followed by recovery in antibiotic-free LB and selection on ampicillin plates. Colonies were screened by PCR using *cyaA*-flanking primers, and successful deletions were confirmed by sequencing and immunoblotting to verify loss of CyaA protein.

### Immunoblotting

To confirm the absence of CyaA protein expression in the Δ*cyaA* mutant, whole-cell lysates were prepared from wild-type, mutant, and complemented strains grown to mid-log phase. Proteins were separated by SDS-PAGE using 4–15% Mini-PROTEAN TG gels (Bio-Rad) and transferred onto PVDF membranes using the Trans-Blot Turb Transfer System (Bio-Rad). Membranes were blocked with 5% skim milk in TBS-T (Tris-buffered saline with 0.1% Tween-20) for 1 hour at room temperature, then incubated overnight at 4°C with rabbit anti-CyaA polyclonal antibody (dilution 1:2,000). After washing, membranes were incubated with HRP-conjugated anti-rabbit IgG secondary antibody (1:10,000) for 1 hour at room temperature. Signal detection was performed using ECL substrate, and images were acquired using the ImageQuant 800 imager (Amersham, UK).

### Sequencing of AJ10 *cyaA* Gene

To analyze potential sequence differences in the *cyaA* gene of the environmental *Vibrio cholerae* AJ10 strain, genomic DNA was extracted using the phenol-chloroform method. The complete *cyaA* open reading frame (~2.5 kb) was amplified using high-fidelity DNA polymerase (Thermo Fisher Scientific) with primers designed to flank the coding region:
F 5′-ATGGCTGAAGTTTACAAAGATG-3′,
R 5′-TTATTATTCGCTCTTTTTCAGC-3′.
The amplification product was verified and purified using a QIAquick PCR Purification Kit (QIAGEN), and submitted to Eurofins Genomics (Tokyo, Japan) for Sanger sequencing. Sequencing reads of amino acid were assembled and aligned using MEGA, BLASTp and Jalview software, and the AJ10 CyaA sequence was compared to

the reference sequence from *V. cholerae* N16961 and known photoactivated cyclases sequence from *Cyanobacteria*, *Oscillatoria*, *Turneriella parva* and *Beggiatoa alba.*

**Heterologous expression in *E. coli* MG1655 Δ*cyaA* Δ*cpdA***

The *Vibrio cholerae cyaA* coding region plus 30 bp of native 5′-UTR was amplified from strain AJ10 genomic DNA with Phusion High-Fidelity polymerase (New England Biolabs, USA) using primer pair:

Vc_EcoRI_F
5′-GAATTCAAGGAGATATACCATGGCTAAAATCAGAGCGTTTATC-3′
Vc_HindIII_R 5′-AAGCTTTTATTTCTTCTTCAACTTGTTCC-3′.

PCR product was digested with EcoRI/HindIII (Takara Bio Inc, Japan) and ligated into similarly digested pBAD33 (Addgene #36267) (13), yielding pBAD-Vc-*cyaA*-His with a C-terminal His6_66 tag (14). For positive control, the *E. coli cyaA* ORF was amplified with primers:

Ec-*cyaA*_EcoRI_F 5′-GAATTCAAGGAGATATACCATGACGACCATTTGCCAAG-3′
Ec-cyaA_HindIII_R 5′-AAGCTTTTATTTCACCTGCCCTGATC-3′

and cloned into pBAD33 to generate pBAD-Ec-cyaA. All inserts were sequence-verified. The plasmids and empty pBAD33 were transformed into *E. coli* MG1655 Δ*cyaA* Δ*cpdA* (Keio alleles JW0123 and JW2669 crossed by P1 transduction) (15, 16). Overnight cultures (LB + 25 µg $ml^{-1}$ chloramphenicol, 30 °C) were diluted 1 : 100 into fresh medium, grown at 30 °C to $OD_{600}$ 0.4, induced with 0.02 % w/v l-arabinose, and shifted to 20 °C for 16 h under dim light. *E. coli* strains expressing AJ10 *cyaA* or empty vector were cultured under $Na^+$ and pH stress conditions (LB ± 200 mM NaCl at pH 8.5), and expression of *nhaA*, *nhaR*, and *rpoS* was analyzed by RT-qPCR as described.

**Construction of the Δ*crp* Deletion Mutant**

The *Vibrio cholerae* AJ10 Δ*crp* (VC2614) mutant was generated via in-frame allelic exchange using the suicide vector pWM91. Briefly, approximately 1,000 bp DNA fragments flanking the upstream and downstream regions of the *crp* gene were amplified from AJ10 genomic DNA using the primer pairs crp-A/crp-B and crp-C/crp-D, respectively (Fig. *S7*). The internal primers (crp-B and crp-C) contained complementary overlapping sequences. The two flanking fragments were joined via overlap extension PCR using the external primers crp-A and crp-D, creating a ~2,000 bp deletion construct.

The resulting fusion product was digested with *SacI* and *XbaI* (Takara Bio Inc, Japan) and ligated into the corresponding sites of pWM91 to generate the mutational plasmid pWM91- Δ*crp* This plasmid was transformed into *E. coli* SM10λpir and subsequently transferred into *V. cholerae* AJ10 via bi-parental conjugation. Initial selection for the first-crossover event (integration of the plasmid into the chromosome) was performed on LB agar supplemented with ampicillin (100 µg/mL) and polymyxin B (50 unit/mL) to select against the *E. coli* donor.

To facilitate the second crossover event and excision of the plasmid backbone, ampicillin-resistant merodiploids were cultured in LB broth without NaCl for 4 hours at 30 °C and then plated onto LB agar containing 10% sucrose. Sucrose-resistant colonies were screened for the loss of ampicillin resistance. The Δ*crp* genotype was confirmed by colony PCR using external primers crp-ext-F and crp-ext-R, and the deletion was further validated by Sanger sequencing of the resulting genomic junction.

**Construction of domain-swap CyaA chimeras**

To investigate the contribution of different regions of CyaA to light responsiveness, domain-swap constructs were generated between the AJ10 and N16961 *cyaA* alleles. Based on sequence alignment (PSIPRED), two junction points were selected: J1, corresponding to the boundary around residues 189/234, and J2, around residues 229/275. These positions were chosen to fall within flexible, non-conserved linker regions to minimize disruption of secondary structure. Using these sites, two chimeras were designed: Chimera A (AJ10 1–189 fused to N16961 229–843) and Chimera B (N16961 1–234 fused to AJ10 275–798). Gene fragments were amplified by high-fidelity PCR (Phusion DNA Polymerase, NEB) using primers listed in Fig. *S2D*. Overlap sequences of ~20-25 bp were incorporated into primer design to enable seamless assembly by Gibson cloning. PCR products were purified with a PCR cleanup kit (Qiagen), quantified, and assembled into the pTrc99A expression vector (Amp^R) linearized with EcoRI/HindIII. Ligation reactions were transformed into *E. coli* DH5α for propagation and sequence-verified by Sanger sequencing (Eurofins Genomics). For functional assays, plasmids carrying AJ10 *cyaA* WT, N16961 *cyaA* WT, Chimera A, or Chimera B were transformed into *E. coli* MG1655 Δ*cyaA* Δ*cpdA* (deficient in endogenous adenylate cyclase and phosphodiesterase) to eliminate background cAMP metabolism. Transformants were grown overnight in LB with 100 µg/mL ampicillin, diluted 1:100 into fresh LB, and cultured at 37 °C with shaking until mid-log phase for cAMP assay.

**CyaA purification and UV-visible spectroscopy**

For protein purification, 500 mL of the induced culture was harvested (4000 ×g, 10 min, 4 °C), resuspended in lysis buffer (20 mM Tris-HCl pH 7.5, 150 mM NaCl, 10 mM imidazole, 1 mM $MgCl_2$, 0.5 mM PMSF, 1 µg $ml^{-1}$ leupeptin) and lysed by high-pressure homogenizer (French Press G-M, Glen Mills, USA) (15000 psi, 4 °C). The clarified lysate (20000 ×g, 30 min) was applied to Ni-NTA resin (Qiagen) pre-equilibrated in lysis buffer; after washing with 30 mM imidazole, His-CyaA was eluted with 250 mM imidazole. Eluate was concentrated and further purified by size-exclusion chromatography (16/60 Superdex 200, GE Healthcare, USA) in 20 mM Tris-HCl pH 7.5, 150 mM NaCl, 1 mM $MgCl_2$. Typical yield was 0.8 ± 0.2 mg per liter with >90 % purity (SDS-PAGE).

Purified protein was adjusted to 10 µM in the SEC buffer and transferred to a 1 cm quartz cuvette. UV-visible absorption spectra (300-600 nm, 1 nm step, 0.5 s integration) were recorded at 25 °C on a UV-vis spectrophotometer (UV-1850, Shimazu, Japan) under dim light (dark spectrum). The same sample was then illuminated in situ for 30 s with a 450 nm LED and immediately rescanned (light spectrum). Difference spectra (Light - Dark) were generated in Prism 10 (Dotmatics, USA). Thermal recovery of the 450 nm absorbance was monitored every 30 s for 10 min after cessation of illumination; data were fitted to a single-exponential decay to obtain τ_½ (17). All assays were repeated with three independent preparations.

**Measurement of Membrane Potential and Sodium Gradient**

Membrane potential (Δψ) and intracellular sodium concentration $[Na^{+}]_i$ were measured using the fluorescent dyes $DiSC_3(5)$ (Invitrogen, USA) and SBFI-AM (Invitrogen), respectively. *V. cholerae* cells were grown to mid-log phase ($OD_{600}$ ≈ 0.5) and resuspended in a buffer containing 50 mM HEPES (pH 7.5), 50 mM NaCl, and 5 mM glucose. Fluorescence was monitored using a spectrophotometer (RF-5300PC, Shimazu, Japan)

For membrane potential measurements, cells were incubated with 2 μM DiSC3(5) for 10 min in the dark to allow dye equilibration. Fluorescence was recorded on a spectrofluorometer (excitation 622 nm; emission 670 nm) as the continuous time course. DiSC3(5) is a lipophilic cation that accumulates in energized cells in proportion to Δψ, resulting in fluorescence quenching; thus, hyperpolarization increases dye accumulation and decreases fluorescence, whereas depolarization increases fluorescence. To convert DiSC3(5) fluorescence to Δψ (mV), we imposed defined $K^+$ diffusion potentials by equilibrating the membrane selectively to $K^+$ with valinomycin. After DiSC3(5) equilibration, valinomycin was added to a final concentration of 10 μM, and the external $K^+$ concentration was adjusted stepwise by adding concentrated KCl while maintaining constant osmolarity by substituting $K^+$ for an inert monovalent cation in the buffer. For each $K^+$ step, fluorescence was allowed to stabilize and the steady-state DiSC3(5) signal was recorded.

For each condition, the expected Δψ associated with the imposed $K^+$ gradient was calculated using the Nernst relationship:

$$\Delta\psi(\mathrm{mV}) = \frac{RT}{F}\ln\left(\frac{[K^+]_\mathrm{o}}{[K^+]_\mathrm{i}}\right) \times 1000$$

where $R$ is the gas constant, $T$ is absolute temperature, and $F$ is Faraday's constant. In these calculations, intracellular $K^+$ was assumed to be constant at $[\mathrm{K}^+]_\mathrm{i}$ = 200 mM, a value commonly used for Gram-negative bacteria under similar conditions (18, 19). Thus, the calibration provides an absolute mV scale for Δψ, while our primary interpretations rely on within-experiment Δψ changes and condition-to-condition comparisons performed using identical buffer and calibration settings. Δψ is reported as inside relative to outside, with more negative values indicating an interior-negative membrane potential.

Radiometric SBFI-AM fluorescence (excitation 340/380 nm; emission 510 nm) was converted to intracellular $Na^+$ concentration, $[Na^+]_\mathrm{i}$, using calibration curves obtained under the same optical settings. To express the $Na^+$ gradient as an electrical equivalent (mV), we calculated the $Na^+$ chemical potential term as:

$$\Delta\mu Na^+(\mathrm{mV}) = \frac{RT}{F}\ln\left(\frac{[Na^+]_\mathrm{o}}{[Na^+]_\mathrm{i}}\right) \times 1000$$

where $R$, $T$ and $F$ are same as above. $[Na^+]_o$was fixed by the assay buffer (50 mM NaCl). Under our conditions, $\frac{RT}{F}$ equals 25.7 mV at room temperature (25°C).

Both parameters were recorded in a time-course manner with alternating light and dim conditions. Baseline values were measured in the dim, followed by light exposure at 0 s and continuous monitoring. Light was then switched off to observe recovery. Fluorescence data were normalized using calibration curves, and membrane potential and sodium gradient values were calculated in mV. Experiments were performed in triplicate and statistical analysis was conducted.

**$Na^+$ Antiporter Inhibition Assay Using EIPA**

Mid-log phase cultures ($OD_{600}$ ≈ 0.5) were divided into three conditions: dim, light, and light + EIPA. $Na^+/H^+$ antiporter inhibitor 5-(N-ethyl-N-isopropyl) amiloride (EIPA) (Cayman Chemical) was added to a final concentration of 25 μM, and cells were incubated at 37°C for 15 minutes prior to illumination. After 1-minute exposure to the respective light conditions, intracellular sodium concentration was measured using the SBFI-AM (Thermo Fisher Scientific). In parallel, swimming speed was quantified under the same light conditions, as previously described.

## Statistical analysis

Statistical tests used for each comparison are specified in the corresponding figure legends. Unless otherwise noted, tests were two-tailed and performed on biological replicate summary values (i.e., movie-level or FOV-level values were first averaged within each biological replicate). Data are presented as mean ± SD or mean ± SEM as indicated. For experiments with two conditions, paired comparisons were evaluated using a two-tailed paired Student's t-test. For experiments with three or more groups, one-way ANOVA followed by Tukey's multiple comparisons test was used. Analyses were performed using OriginPro and MATLAB with a significance threshold of $P < 0.05$ (*) and $P < 0.01$ (**).

## Supplementary References:


1. M. J. Schnitzer, Theory of continuum random walks and application to chemotaxis. *Phys. Rev. E* **48**, 2553–2568 (1993).
2. E. A. Codling, M. J. Plank, S. Benhamou, Random walk models in biology. *J. R. Soc. Interface* **5**, 813–834 (2008).
3. M. IWANAGA, *et al.*, Characteristic of Vibrio cholerae O1 Isolated in the Aja River. *Journal of the Japanese Association for Infectious Diseases* **59**, 551–558 (1985).
4. Y. Wang, *et al.*, Tidal Variability of Phytoplankton Distribution in the Highly Turbid Changjiang River Estuary: Mechanisms and Implications. *J. Geophys. Res. Oceans* **128** (2023).
5. J. T. O. Kirk, *Light and photosynthesis in aquatic ecosystems* (Cambridge University Press, 1994).
6. J. Schindelin, *et al.*, Fiji: an open-source platform for biological-image analysis. *Nat. Methods* **9**, 676–682 (2012).
7. J.-Y. Tinevez, *et al.*, TrackMate: An open and extensible platform for single-particle tracking. *Methods* **115**, 80–90 (2017).
8. G. Frangipane, *et al.*, Dynamic density shaping of photokinetic E. coli. *Elife* **7** (2018).
9. P. Luo, T. Su, C. Hu, C. Ren, A Novel and Simple PCR Walking Method for Rapid Acquisition of Long DNA Sequence Flanking a Known Site in Microbial Genome. *Mol. Biotechnol.* **47**, 220–228 (2011).
10. N. Philippe, J.-P. Alcaraz, E. Coursange, J. Geiselmann, D. Schneider, Improvement of pCVD442, a suicide plasmid for gene allele exchange in bacteria. *Plasmid* **51**, 246–255 (2004).
11. R. C. Molina-Quiroz, A. Camilli, C. A. Silva-Valenzuela, "Role of Bacteriophages in the Evolution of Pathogenic Vibrios and Lessons for Phage Therapy" in (2023), pp. 149–173.
12. M. S. Donnenberg, J. B. Kaper, Construction of an eae deletion mutant of enteropathogenic Escherichia coli by using a positive-selection suicide vector. *Infect. Immun.* **59**, 4310–4317 (1991).
13. L. M. Guzman, D. Belin, M. J. Carson, J. Beckwith, Tight regulation, modulation, and high-level expression by vectors containing the arabinose PBAD promoter. *J. Bacteriol.* **177**, 4121–4130 (1995).
14. K. Terpe, Overview of tag protein fusions: from molecular and biochemical fundamentals to commercial systems. *Appl. Microbiol. Biotechnol.* **60**, 523–533 (2003).
15. L. C. Thomason, N. Costantino, D. L. Court, *E. coli* Genome Manipulation by P1 Transduction. *Curr. Protoc. Mol. Biol.* **79** (2007).

16. T. Baba, *et al.*, Construction of *Escherichia coli* K-12 in-frame, single-gene knockout mutants: the Keio collection. *Mol. Syst. Biol.* **2** (2006).
17. M. Gauden, *et al.*, Hydrogen-bond switching through a radical pair mechanism in a flavin-binding photoreceptor. *Proceedings of the National Academy of Sciences* **103**, 10895–10900 (2006).
18. R. D. Kitko, J. C. Wilks, G. M. Garduque, J. L. Slonczewski, Osmolytes Contribute to pH Homeostasis of Escherichia coli. *PLoS One* **5**, e10078 (2010).
19. J. Stautz, *et al.*, Molecular Mechanisms for Bacterial Potassium Homeostasis. *J. Mol. Biol.* **433**, 166968 (2021).
